# Supplementary material for: What can be learned from fishers’ perceptions for fishery management planning? Case study insights from Sainte-Marie, Madagascar
Source: PLoS One. 2021 Nov 15;16(11):e0259792. doi: 10.1371/journal.pone.0259792 (PMC8592436; doi:10.1371/journal.pone.0259792)
Supplement: S1 Table — In the local fishing vocabulary, common names were often applied to multiple species of fishes. For this reason, we indicate the most representative English fish family, associated with English species name associated with the common Malagasy name. (DOCX) [file pone.0259792.s002.docx]

|  |  |  | **Proportion of the fishermen (%)** | | | | | |
| --- | --- | --- | --- | --- | --- | --- | --- | --- |
| **Local name** | **Family** | **Species** | **Total** | **Hook** | **Nets** | **Diving** | **Traps** | **Foot** |
| Lagnoran | Carangidae | *Caranx sp., Carangoides sp.* | 43 | 51 | 37 | 12 | - | - |
| Femalandy | Siganidae | *Siganus sp.* | 26 | 8 | 31 | 27 | 35 | - |
| Pêche cavale | Carangidae | *Selar crumenophtalmus* | 25 | 64 | 36 | - | - | - |
| Fiambazan | Scaridae | *Scarus sp., Chlorurus sp., Cetoscarus sp., Leptoscarus sp., Calotomus sp.* | 23 | 4 | 9 | 52 | 35 | - |
| Mandrantongo | Lethrinidae | *Lethrinus harak* | 21 | 48 | 33 | 19 | - | - |
| Menaeliky | Lethrinidae | *Lethrinus crocineus* | 20 | 100 | - | - | - | - |
| Cabot | Serranidae | *Epinephelus sp., Cephalopholis sp.* | 17 | 39 | 11 | 50 | - | - |
| Mondrazy | Mullidae | *Parupeneus sp.* | 17 | 24 | 47 | 18 | 12 | - |
| Orita | Octopodidae | *Octopus cyanea* | 15 | - | - | 80 | - | 20 |
| Bonite | Scombridae | *Euthynnus affinis, Katsuwonus pelamis* | 14 | 14 | 86 | - | - | - |
| Saroron | Loliginidae | *Sepioteuthis sp.* | 13 | 100 | - | - | - | - |
| Ankasera | Sphyraenidae | *Sphyraena sp.* | 11 | 73 | 27 | - | - | - |
| Orandretsy | Palinuridae | *Palinurus sp.* | 10 | - | - | 100 | - | - |
