# Supplementary material for: What can be learned from fishers’ perceptions for fishery management planning? Case study insights from Sainte-Marie, Madagascar
Source: PLoS One. 2021 Nov 15;16(11):e0259792. doi: 10.1371/journal.pone.0259792 (PMC8592436; doi:10.1371/journal.pone.0259792)
Supplement: S2 Table — (DOCX) [file pone.0259792.s003.docx]

| **Topic** | **General questions** |
| --- | --- |
| Socioeconomic and demographic characteristics | How old are you?  How long have you been fishing for? |
|  | What gear do you use? |
|  | What gear do you own?  What do you do with your catches? |
|  | Do you belong to any association? |
|  | Do you have other sources of income? |
|  | What is a normal income for a month?  What is your education level? |
| Perceptions | Have you noticed any changes in fishing sites?  At what distance from the shore did you fish when you started?  At what distance from the shore do you fish now?  Have you noticed any changes in the number of fish?  Have you noticed the absence of known species in the area?  Have you noticed the presence of novel species in the area?  Have you noticed any changes in the size of the fish?  For 3 target species: what was a good catch over the past year (2016-2017)? The year before the cyclone Yvan (2007-2008)? And/or when you started to fish? *  Has your fishing effort changed? |
|  | What could cause the changes you observed? |
|  | If there was a hypothetical decline of 50%, what would you do?  What could be done to reduce the ecological changes? |
| Additional questions | Would you recommend your children to be fishers? |
|  | Do you know any customary rules related to natural resource management (fady or dina)?  Are you married? Do you have children? |
