# Supplementary material for: What can be learned from fishers’ perceptions for fishery management planning? Case study insights from Sainte-Marie, Madagascar
Source: PLoS One. 2021 Nov 15;16(11):e0259792. doi: 10.1371/journal.pone.0259792 (PMC8592436; doi:10.1371/journal.pone.0259792)
Supplement: S3 Table — (DOCX) [file pone.0259792.s004.docx]

| **Village** | **Borough** | **Men interviewed** | **Women interviewed** | **Total interviewed** |
| --- | --- | --- | --- | --- |
| Ambatouro | Ambatourao | 9 | 1 | 10 |
| Ambodiatafana |  | 4 | - | 4 |
| Ifotatra |  | 5 | 1 | 6 |
| Ambodifotatra | Ambodifotatra | 8 | 1 | 9 |
| Analaradjy |  | 3 | - | 3 |
| Ankobaoba |  | 10 | 2 | 12 |
| St_joseph |  | 6 | 2 | 8 |
| Agnafiafy | Loukintsy | 4 | 2 | 6 |
| Anivorano |  | 4 | 1 | 5 |
| Loukintsy |  | 7 | - | 7 |
| Maromandia |  | 6 | - | 6 |
| Sahasifotra |  | 8 | 1 | 8 |
| Agniribe | Vohilava | 9 | 1 | 9 |
| Ambodiforaha |  | 7 | 2 | 7 |
| Mahavelo |  | 8 | 2 | 8 |
| Vohilava |  | 11 | 2 | 11 |
|  |  | 109 | 18 | 127 |
