# Supplementary material for: What can be learned from fishers’ perceptions for fishery management planning? Case study insights from Sainte-Marie, Madagascar
Source: PLoS One. 2021 Nov 15;16(11):e0259792. doi: 10.1371/journal.pone.0259792 (PMC8592436; doi:10.1371/journal.pone.0259792)
Supplement: S4 Table — Sp = species, df = degrees of freedom, REML = log-restricted likelihood, Dev. Expl = Deviance explained. (DOCX) [file pone.0259792.s005.docx]

| **Model** | **Specification** | **df** | **AIC** | **REML** | **R^2^** | **Dev. expl** |
| --- | --- | --- | --- | --- | --- | --- |
| **Mod1** | Sp. family + s(time, by sp. family) | 130.86 | 4566.63 | -2344.65 | 0.757 | 0.814 |
| **Mod2** | Sp. family + s(time) | 105.97 | 4521.63 | -2333.55 | 0.751 | 0.804 |
| **Mod3** | Sp. family | 98.21 | 4627.77 | -2370.79 | 0.709 | 0.763 |
| **Mod4** | s(time) | 95.65 | 4704.91 | 2422.05 | 0.592 | 0.725 |
