# Supplementary material for: What can be learned from fishers’ perceptions for fishery management planning? Case study insights from Sainte-Marie, Madagascar
Source: PLoS One. 2021 Nov 15;16(11):e0259792. doi: 10.1371/journal.pone.0259792 (PMC8592436; doi:10.1371/journal.pone.0259792)
Supplement: S5 Table — (DOCX) [file pone.0259792.s006.docx]

| **Model** | **Specification** | **AIC** | **R^2^** |
| --- | --- | --- | --- |
| **Mod1b** | Gear + year by gear | 732274.4 | 0.229 |
| **Mod2b** | Gear + year | 738363.5 | 0.223 |
| **Mod3b** | Gear | 802406.8 | 0.155 |
| **Mod4b** | Year | 875276.7 | 0.078 |
