# Supplementary material for: What can be learned from fishers’ perceptions for fishery management planning? Case study insights from Sainte-Marie, Madagascar
Source: PLoS One. 2021 Nov 15;16(11):e0259792. doi: 10.1371/journal.pone.0259792 (PMC8592436; doi:10.1371/journal.pone.0259792)
Supplement: S6 Table — (DOCX) [file pone.0259792.s007.docx]

| **Common names** | **Number of citations** | **Note** |
| --- | --- | --- |
| Dugong | 23 | Mammal |
| Enamahely | 23 | *Mullidae* |
| Antafan | 20 | *Mullidae* |
| Concombre de mer | 8 | Echinoderm |
| Angara | 4 |  |
| Ophambe | 4 |  |
| Fiamandry | 3 |  |
| Menavalo | 3 |  |
| Tortue de mer | 3 | Reptile |
| Aleta | 2 |  |
| Ambitry | 2 |  |
| Amboso | 2 |  |
| Ancarangua | 2 |  |
| Espadon | 2 |  |
| Madame Tombee | 2 |  |
| Vaoho | 2 |  |
| Varvaran | 2 |  |
| Antalanta | 1 |  |
| Dents de chien | 1 |  |
| Lotsabatra | 1 |  |
| Marie-Therese | 1 |  |
| Matibango | 1 |  |
| Menailk | 1 |  |
| Merou | 1 |  |
| Pascavale | 1 |  |
| Sarde | 1 |  |
| kind of carangue | 1 |  |
| Tangorongoro | 1 |  |
| Tsetseko | 1 |  |
| Thon Blanc | 1 |  |
| Requin | 1 |  |
