# Supplementary material for: What can be learned from fishers’ perceptions for fishery management planning? Case study insights from Sainte-Marie, Madagascar
Source: PLoS One. 2021 Nov 15;16(11):e0259792. doi: 10.1371/journal.pone.0259792 (PMC8592436; doi:10.1371/journal.pone.0259792)
Supplement: S7 Table — KMO > 5 are considered well-sampled; variables with a KMO < could be suspect or have low variance, and should therefore be interpreted with caution. In the case of the number of fishes, fishers almost all indicated a decline. Bartlett’s test was significant (p<0.001), which indicate that our variables were related and therefore suitable for a factor analysis. (DOCX) [file pone.0259792.s008.docx]

| **Variable** | **MSA** |
| --- | --- |
| Restrictions | 0.8 |
| Causes | 0.76 |
| Fishing distance | 0.72 |
| Disapearance | 0.68 |
| Leisure | 0.67 |
| Gender | 0.65 |
| Hotels | 0.61 |
| Association | 0.61 |
| Fish size | 0.6 |
| Lagoons | 0.59 |
| Attachment | 0.57 |
| Coping (realized) | 0.57 |
| Shops | 0.56 |
| Age | 0.47 |
| Dependence | 0.46 |
| Coping (hypothetical) | 0.46 |
| Number of fishes | 0.37 |
| **Overall MSA** | **0.61** |
