# Supplementary material for: What can be learned from fishers’ perceptions for fishery management planning? Case study insights from Sainte-Marie, Madagascar
Source: PLoS One. 2021 Nov 15;16(11):e0259792. doi: 10.1371/journal.pone.0259792 (PMC8592436; doi:10.1371/journal.pone.0259792)
Supplement: S8 Table — (DOCX) [file pone.0259792.s009.docx]

|  | Dim.1 | Dim.2 | Dim.3 | Dim.4 | Dim.5 |
| --- | --- | --- | --- | --- | --- |
| Local assets | 3.48 | 32.63 | 5.08 | 4.63 | 42.09 |
| Individual attributes | 26.30 | 29.49 | 26.66 | 27.58 | 36.24 |
| Ecological health | 25.32 | 13.03 | 44.91 | 7.42 | 10.29 |
| Coping | 26.35 | 17.50 | 13.78 | 58.20 | 5.42 |
| Causes | 18.55 | 7.34 | 9.57 | 2.17 | 5.97 |
