# Supplementary material for: What can be learned from fishers’ perceptions for fishery management planning? Case study insights from Sainte-Marie, Madagascar
Source: PLoS One. 2021 Nov 15;16(11):e0259792. doi: 10.1371/journal.pone.0259792 (PMC8592436; doi:10.1371/journal.pone.0259792)
Supplement: S9 Table — (DOCX) [file pone.0259792.s010.docx]

| Variable |  | R^2^ |
| --- | --- | --- |
|  | Gender | 0.52 |
|  | Score_Causes | 0.39 |
|  | Score_FishDisp | 0.34 |
|  | Score_CopingH | 0.36 |
|  | Score_CopR | 0.36 |
|  | Score_FishDist | 0.29 |
|  | Score_Solution | 0.20 |
|  | Association | 0.19 |
|  | Score_FishSiz | 0.12 |
|  | Leisure | 0.07 |
|  | Attachment | 0.05 |
|  | Tourism | 0.05 |
| Category |  | Estimate |
|  | Gender=IN_woman | 1.51 |
|  | Score_Causes=Co_notfish | 1.21 |
|  | Score_FishDisp=ED_dispno | 0.84 |
|  | Score_CopingH=CH_continue | 1.47 |
|  | Score_FishDist=ED_distno | 0.78 |
|  | Score_Solution=RE_no | 0.67 |
|  | Score_CopR=CR_decrease | 0.65 |
|  | Association=IN_Assono | 0.68 |
|  | Score_FishSiz=ED_sizeno | 0.51 |
|  | Score_CopR=CR_continue | 0.54 |
|  | Leisure=LA_leisno | 0.38 |
|  | Attachment=IN_Attno | 0.37 |
|  | Tourism=LA_hotno | 0.31 |
|  | Tourism=LA_hot | -0.31 |
|  | Attachment=IN_att | -0.37 |
|  | Leisure=LA_leis | -0.38 |
|  | Score_FishSiz=ED_Size | -0.51 |
|  | Score_CopingH=CH_adapt | -1.12 |
|  | Association=IN_Asso | -0.68 |
|  | Score_Solution=RE_yes | -0.67 |
|  | Score_FishDist=ED_dist | -0.78 |
|  | Score_FishDisp=ED_disp | -0.84 |
|  | Score_CopR=CR_adapt | -1.19 |
|  | Score_Causes=Co_fishing | -1.21 |
|  | Gender=IN_man | -1.51 |
