# Supplementary material for: What can be learned from fishers’ perceptions for fishery management planning? Case study insights from Sainte-Marie, Madagascar
Source: PLoS One. 2021 Nov 15;16(11):e0259792. doi: 10.1371/journal.pone.0259792 (PMC8592436; doi:10.1371/journal.pone.0259792)
Supplement: S10 Table — (DOCX) [file pone.0259792.s011.docx]

| Variable |  | R^2^ |
| --- | --- | --- |
|  | Lagoons | 0.38 |
|  | Shops | 0.37 |
|  | Leisure | 0.20 |
|  | Age | 0.20 |
|  | Score_CopR | 0.20 |
|  | Score_FishSiz | 0.18 |
|  | Attachment | 0.13 |
|  | Gender | 0.11 |
|  | Score_Causes | 0.10 |
|  | Income.div | 0.10 |
|  | Score_CopingH | 0.12 |
|  | Score_FishAb | 0.08 |
|  | Association | 0.06 |
|  | Tourism | 0.04 |
| Category |  | Estimate |
|  | Lagoons=LA_lag | 0.79 |
|  | Shops=LA_sho | 0.73 |
|  | Leisure=LA_leis | 0.54 |
|  | Age=IN_young | 0.53 |
|  | Score_FishSiz=ED_sizeno | 0.50 |
|  | Score_CopR=CR_continue | 1.01 |
|  | Attachment=IN_att | 0.49 |
|  | Score_CopingH=CH_continue | 0.66 |
|  | Gender=IN_woman | 0.57 |
|  | Score_Causes=Co_fishing | 0.52 |
|  | Income.div=IN_dep | 0.38 |
|  | Score_FishAb=ED_Numno | 0.73 |
|  | Association=IN_Asso | 0.31 |
|  | Tourism=LA_hot | 0.23 |
|  | Tourism=LA_hotno | -0.23 |
|  | Association=IN_Assono | -0.31 |
|  | Score_CopingH=CH_decrease | -0.42 |
|  | Score_CopR=CR_decrease | -0.80 |
|  | Score_FishAb=ED_Num | -0.73 |
|  | Income.div=IN_depno | -0.38 |
|  | Score_Causes=Co_notfish | -0.52 |
|  | Gender=IN_man | -0.57 |
|  | Attachment=IN_Attno | -0.49 |
|  | Score_FishSiz=ED_Size | -0.50 |
|  | Age=IN_old | -0.53 |
|  | Leisure=LA_leisno | -0.54 |
|  | Shops=LA_shono | -0.73 |
|  | Lagoons=LA_lagno | -0.79 |
