# Supplementary material for: What can be learned from fishers’ perceptions for fishery management planning? Case study insights from Sainte-Marie, Madagascar
Source: PLoS One. 2021 Nov 15;16(11):e0259792. doi: 10.1371/journal.pone.0259792 (PMC8592436; doi:10.1371/journal.pone.0259792)
Supplement: S11 Table — (DOCX) [file pone.0259792.s012.docx]

| Variable |  | R^2^ |
| --- | --- | --- |
|  | Score_FishAb | 0.38 |
|  | Age | 0.31 |
|  | Score_CopR | 0.18 |
|  | Score_FishDist | 0.12 |
|  | Score_FishSiz | 0.11 |
|  | Score_FishDisp | 0.11 |
|  | Score_Causes | 0.11 |
|  | Gender | 0.09 |
|  | Lagoons | 0.07 |
| Category |  | Estimate |
|  | Score_FishAb=ED_Numno | 1.38 |
|  | Age=IN_young | 0.59 |
|  | Score_CopR=CR_continue | 0.83 |
|  | Score_FishDist=ED_dist | 0.37 |
|  | Score_FishSiz=ED_Size | 0.36 |
|  | Score_FishDisp=ED_dispno | 0.36 |
|  | Score_Causes=Co_notfish | 0.47 |
|  | Gender=IN_man | 0.46 |
|  | Lagoons=LA_lagno | 0.31 |
|  | Lagoons=LA_lag | -0.31 |
|  | Score_CopR=CR_decrease | -0.67 |
|  | Gender=IN_woman | -0.46 |
|  | Score_Causes=Co_fishing | -0.47 |
|  | Score_FishDisp=ED_disp | -0.36 |
|  | Score_FishSiz=ED_sizeno | -0.36 |
|  | Score_FishDist=ED_distno | -0.37 |
|  | Age=IN_old | -0.59 |
|  | Score_FishAb=ED_Num | -1.38 |
