# Supplementary material for: What can be learned from fishers’ perceptions for fishery management planning? Case study insights from Sainte-Marie, Madagascar
Source: PLoS One. 2021 Nov 15;16(11):e0259792. doi: 10.1371/journal.pone.0259792 (PMC8592436; doi:10.1371/journal.pone.0259792)
Supplement: S12 Table — (DOCX) [file pone.0259792.s013.docx]

| Variable |  | R2 |
| --- | --- | --- |
|  | Score_CopingH | 0.63 |
|  | Income.div | 0.30 |
|  | Score_CopR | 0.14 |
|  | Score_FishDist | 0.06 |
|  | Lagoons | 0.06 |
|  | Attachment | 0.05 |
|  | Gender | 0.05 |
|  | Score_FishAb | 0.04 |
|  | Shops | 0.03 |
| Category |  | Estimate |
|  | Score_CopingH=CH_adapt | 0.88 |
|  | Income.div=IN_dep | 0.55 |
|  | Score_CopR=CR_adapt | 0.52 |
|  | Score_FishDist=ED_dist | 0.26 |
|  | Score_CopingH=CH_continue | 0.14 |
|  | Lagoons=LA_lagno | 0.27 |
|  | Attachment=IN_Attno | 0.25 |
|  | Gender=IN_woman | 0.32 |
|  | Score_FishAb=ED_Num | 0.45 |
|  | Shops=LA_shono | 0.19 |
|  | Shops=LA_sho | -0.19 |
|  | Score_FishAb=ED_Numno | -0.45 |
|  | Gender=IN_man | -0.32 |
|  | Attachment=IN_att | -0.25 |
|  | Lagoons=LA_lag | -0.27 |
|  | Score_FishDist=ED_distno | -0.26 |
|  | Score_CopR=CR_decrease | -0.32 |
|  | Income.div=IN_depno | -0.55 |
|  | Score_CopingH=CH_decrease | -1.02 |
