# Supplementary material for: What can be learned from fishers’ perceptions for fishery management planning? Case study insights from Sainte-Marie, Madagascar
Source: PLoS One. 2021 Nov 15;16(11):e0259792. doi: 10.1371/journal.pone.0259792 (PMC8592436; doi:10.1371/journal.pone.0259792)
Supplement: S13 Table — (DOCX) [file pone.0259792.s014.docx]

| Variable |  | R^2^ |
| --- | --- | --- |
|  | Tourism | 0.40 |
|  | Attachment | 0.32 |
|  | Shops | 0.18 |
|  | Lagoons | 0.17 |
|  | Age | 0.11 |
|  | Score_FishDist | 0.10 |
|  | Score_Causes | 0.05 |
|  | Score_CopR | 0.06 |
| Category |  | Estimate |
|  | Tourism=LA_hot | 0.60 |
|  | Attachment=IN_Attno | 0.59 |
|  | Shops=LA_sho | 0.40 |
|  | Lagoons=LA_lag | 0.42 |
|  | Age=IN_old | 0.31 |
|  | Score_FishDist=ED_dist | 0.30 |
|  | Score_CopR=CR_continue | 0.40 |
|  | Score_Causes=Co_notfish | 0.29 |
|  | Score_Causes=Co_fishing | -0.29 |
|  | Score_FishDist=ED_distno | -0.30 |
|  | Age=IN_young | -0.31 |
|  | Lagoons=LA_lagno | -0.42 |
|  | Shops=LA_shono | -0.40 |
|  | Attachment=IN_att | -0.59 |
|  | Tourism=LA_hotno | -0.60 |
