# Supplementary material for: What can be learned from fishers’ perceptions for fishery management planning? Case study insights from Sainte-Marie, Madagascar
Source: PLoS One. 2021 Nov 15;16(11):e0259792. doi: 10.1371/journal.pone.0259792 (PMC8592436; doi:10.1371/journal.pone.0259792)
Supplement: S1 File — (PDF) [file pone.0259792.s015.pdf]

Supplementary file S1: R scripts for GAM and GLM

Written by: Thaïs Bernos

1: Catch sizes

We used generalized additive models (GAMs) to model temporal changes in best catch sizes for 13 of the most frequent target species family (local Malagasy name) identified from the interviews. GAMs were ideally suited to the structure of our data and the nature of our analysis because; 1) their non-parametric smoothing function (hereafter referred to as smoothers) allowed us to model nonlinear temporal trends (62,63); 2) they can incorporate both continuous and categorical variables; 3) they can accommodate random effects; and, 4) they estimate the shape of the relationship from the data itself (we did not have to specify any a-priori shape). For these reasons, GAM represented a flexible and powerful approach to model temporal trends in best catches, as well as their nature and timing.

For this analysis, you will need the following packages:

```
library(mgcv) #GAM fitting
library(ggplot2) # plots
library(mgcViz) #plotting GAM
```

1.1 Load data

Here, we will use the "Catches.txt" dataset. In the code below, I named it ten_main.

```
head(ten_main)
```

```
##    Fisher_ID malagasy Nom_courant       Family     Genus             species
## 59        73 Ankasera   Barracuda Sphyraenidae Sphyraena Sphyraena_barracuda
## 60        73 Ankasera   Barracuda Sphyraenidae Sphyraena Sphyraena_barracuda
## 61        27 Ankasera   Barracuda Sphyraenidae Sphyraena Sphyraena_barracuda
## 62        61 Ankasera   Barracuda Sphyraenidae Sphyraena Sphyraena_barracuda
## 63        68 Ankasera   Barracuda Sphyraenidae Sphyraena Sphyraena_barracuda
## 64        83 Ankasera   Barracuda Sphyraenidae Sphyraena Sphyraena_barracuda
##    Quantite Year Technique
## 59      150 1997      Hook
## 60      150 2007      Hook
## 61       60 2012      Hook
## 62       60 2007    Filets
## 63       60 2007      Hook
## 64       60 2007    Filets
```

1.2 GAM fitting

We use negative binomial GAMs and a logit link. A first model (Mod1) included different intercept for each species, a smoother for time, and its interaction with species. As perceptions can vary among fishermen, we fit fisherman identity as a random intercept and slope. To investigate plausible alternative hypotheses, we also construct several additional candidate models:

- Mod2 included the species intercept and a smoother for time.
- Mod3 includes the species intercept.

- Mod4 includes the time smoother.

```
#with specie sintercept, time smoother, interaction between the two, random slope+intercept
Mod1 <- gam(Quantite~malagasy+s(Year,by=malagasy) +s(Fisher_ID, bs="re")+s(Fisher_ID, Year, bs="re"),
            data=ten_main,method="REML",family=nb()) #with random
#with species intercept, time smoother, and random slope+intercept
Mod2 <- gam(Quantite~malagasy+s(Year) +s(Fisher_ID, bs="re")+s(Fisher_ID, Year, bs="re"),
            data=ten_main,method="REML",family=nb()) #with random
#with species intercept, time linear, and random slope + intercept
Mod3 <- gam(Quantite~malagasy+s(Fisher_ID, bs="re")+s(Fisher_ID, Year, bs="re"),
            data=ten_main,method="REML",family=nb()) #with random
Mod4 <- gam(Quantite~s(Year) +s(Fisher_ID, bs="re")+s(Fisher_ID, Year, bs="re"),
            data=ten_main,method="REML",family=nb()) #with random
```

1.3 GAM Model selection

We identify the best model as the one with; the lowest AIC criterion, highest restricted log-likelihood, and highest explanatory power.

```
modAIC <- AIC(Mod1,Mod2,Mod3,Mod4)
modAIC$spcrit <- c(summary(Mod1)$sp.criterion,summary(Mod2)$sp.criterion,summary(Mod3)$sp.criterion,
                   summary(Mod4)$sp.criterion)
modAIC$rsq <- c(summary(Mod1)$r.sq,summary(Mod2)$r.sq,summary(Mod3)$r.sq,summary(Mod4)$r.sq)
modAIC$dexp<- c(summary(Mod1)$dev.expl,summary(Mod2)$dev.expl,summary(Mod3)$dev.expl,
                summary(Mod4)$dev.expl)
modAIC
```

```
##             df      AIC   spcrit        rsq       dexp
## Mod1 39.737631 4958.133 2477.603 0.16267807 0.48032022
## Mod2 19.313170 4936.808 2471.420 0.16463800 0.46278094
## Mod3 14.979450 4986.506 2493.368 0.16609875 0.40867742
## Mod4  9.163456 5268.838 2641.140 0.01742669 0.05915808
```

Furthermore, we can evaluate the significance of the fixed effect using Wald's test.

```
anova.gam(Mod1,Mod2,test="F")# the interaction is not significant.
```

```
## Analysis of Deviance Table
##
## Model 1: Quantite ~ malagasy + s(Year, by = malagasy) + s(Fisher_ID, bs = "re") +
##     s(Fisher_ID, Year, bs = "re")
## Model 2: Quantite ~ malagasy + s(Year) + s(Fisher_ID, bs = "re") + s(Fisher_ID,
##     Year, bs = "re")
##   Resid. Df Resid. Dev     Df Deviance Pr(>Chi)
## 1    519.65     4878.7
## 2    542.82     4898.2 -23.17  -19.524   0.6798
```

```
anova.gam(Mod2,Mod3,test="F")
```

```
## Analysis of Deviance Table
##
## Model 1: Quantite ~ malagasy + s(Year) + s(Fisher_ID, bs = "re") + s(Fisher_ID,
##     Year, bs = "re")
## Model 2: Quantite ~ malagasy + s(Fisher_ID, bs = "re") + s(Fisher_ID,
```

```
##     Year, bs = "re")
##   Resid. Df Resid. Dev      Df Deviance  Pr(>Chi)
## 1    542.82     4898.2
## 2    547.90     4956.5 -5.0732  -58.365 2.919e-11 ***
## ---
## Signif. codes:  0 '***' 0.001 '**' 0.01 '*' 0.05 '.' 0.1 ' ' 1
```

```
anova.gam(Mod2,Mod4,test="F")
```

```
## Analysis of Deviance Table
##
## Model 1: Quantite ~ malagasy + s(Year) + s(Fisher_ID, bs = "re") + s(Fisher_ID,
##     Year, bs = "re")
## Model 2: Quantite ~ s(Year) + s(Fisher_ID, bs = "re") + s(Fisher_ID, Year,
##     bs = "re")
##   Resid. Df Resid. Dev      Df Deviance  Pr(>Chi)
## 1    542.82     4898.2
## 2    552.79     5250.5 -9.9612  -352.33 < 2.2e-16 ***
## ---
## Signif. codes:  0 '***' 0.001 '**' 0.01 '*' 0.05 '.' 0.1 ' ' 1
```

1.4 GAM model diagnosis

```
gam.check(Mod2)
```

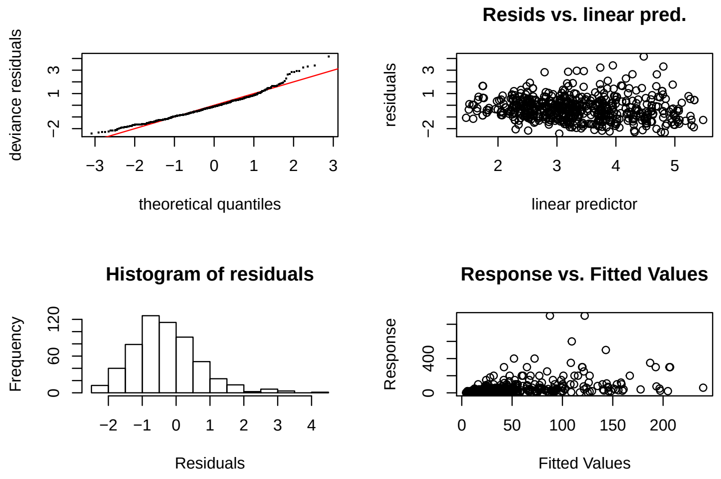


```
##
## Method: REML   Optimizer: outer newton
## full convergence after 7 iterations.
## Gradient range [-0.00515281,0.0001353548]
## (score 2471.42 & scale 1).
## Hessian positive definite, eigenvalue range [0.0003647243,299.3576].
## Model rank =  24 / 24
##
## Basis dimension (k) checking results. Low p-value (k-index<1) may
## indicate that k is too low, especially if edf is close to k'.
##
```

```
##                        k'    edf k-index p-value
## s(Year)            9.0000 3.5636    0.59  <2e-16 ***
## s(Fisher_ID)       1.0000 0.0103    0.50  <2e-16 ***
## s(Fisher_ID,Year) 1.0000 0.8769    0.70  <2e-16 ***
## ---
## Signif. codes:  0 '***' 0.001 '**' 0.01 '*' 0.05 '.' 0.1 ' ' 1
```

The plots show that the model fits well, but K is low. Let's try to increase K and see if we can get p > 0.05. However, when increasing k, edf and k don't really change. This shows that the model is fine; for this reason, default k (k=10) is fine.

1.5 Plot GAM

```
#convert the gam to the gamviz class
b<- getViz(Mod2)
o <- plot(sm(b,1)) # this plots the first smooth component

plot(sm(b,1)) + l_fitLine(colour = "red",size=2) + #this adds the fitline
  l_rug(mapping = aes(x=x, y=y), alpha = 0.8) + #add rugs on x and y axes
  l_ciLine(level=0.95, colour = "blue", linetype = 2,size=2) + # 95% confidence intervals
  l_points(shape = 21, size = 3) +
  ylim(-1,1.5)+
  theme(axis.text=element_text(size=20,face="bold"),
        axis.title=element_text(size=24,face="bold"),
        panel.grid.major.y = element_blank(),
        panel.grid.major.x = element_line(colour = "grey"))
```

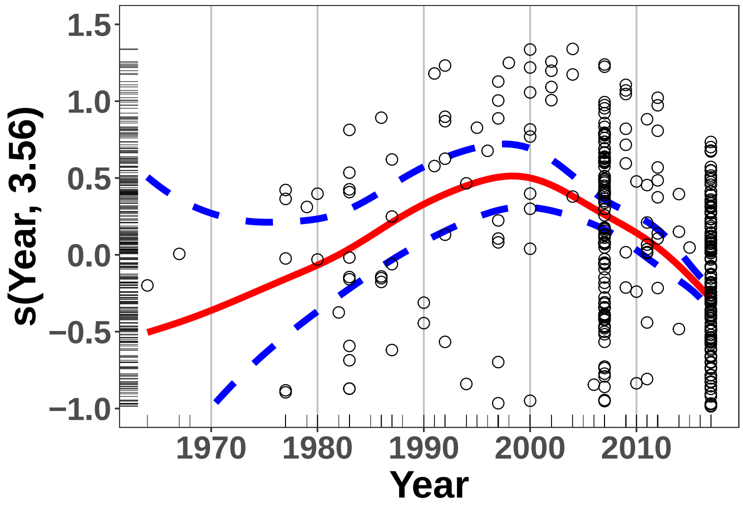

2. Fishing site locations

Here, we used generalized linear models (GLM) to model temporal changes in best catch sizes for 13 of the most frequent target species family (local Malagasy name) identified from the interviews.

2.1 Load data

Here, we will use the "Distance.txt" dataset. In the code below, I named it Fi.

```
head(Fi)
```

```
##   ID TechP  Temps Date Dist.m
## 1  1  Hook Before 1971    400
## 2  1  Hook  After 2017   5000
## 3  2   Net Before 2003    150
## 4  2   Net  After 2017   1000
## 5  3  Hook Before 2010    150
## 6  3  Hook  After 2017   1000
```

```
#Organize levels for plotting later
Fi$TechP=factor(Fi$TechP,levels=c("Hook","Net","Diving","Trap"))
```

2.2 GLM fitting

To model temporal changes in fishing distance from the shore, we specified a poisson distribution and a log link. As the magnitude of the perceived changes could vary by gear, we fitted a first model (Mod1b) with an interaction between fishing gear and time as a fixed effect. Mod2b included an intercept for gear and time; Mod 3 and 4 included either one of the gear intercept or time.

```
M1 <- glm(Dist.m~  Date*TechP,family=poisson,data=Fi)#**
M2 <- glm(Dist.m~ Date + TechP, family=poisson,data=Fi)
M3 <- glm(Dist.m~ TechP, family=poisson,data=Fi)
M4 <- glm(Dist.m~ Date , family=poisson,data=Fi)
```

2.3 GLM selection

We identified the best model based on the lowest AIC and the variance explained.

```
AIC(M1,M2,M3,M4)
```

```
##    df      AIC
## M1  8 732274.4
## M2  5 738363.5
## M3  4 802406.8
## M4  2 875276.7
```

```
with(summary(M1), 1 - deviance/null.deviance)
```

```
## [1] 0.2291898
```

```
with(summary(M2), 1 - deviance/null.deviance)
```

```
## [1] 0.2227572
```

```r
with(summary(M3), 1 - deviance/null.deviance)
```

```
## [1] 0.1551653
```

```r
with(summary(M4), 1 - deviance/null.deviance)
```

```
## [1] 0.07825606
```

Use likelihood ratio test to test the significance of each fixed effects.

```r
anova(M1,M2,test="Chisq")
```

```
## Analysis of Deviance Table
##
## Model 1: Dist.m ~ Date * TechP
## Model 2: Dist.m ~ Date + TechP
##   Resid. Df Resid. Dev Df Deviance  Pr(>Chi)
## 1       199     730366
## 2       202     736461 -3    -6095 < 2.2e-16 ***
## ---
## Signif. codes:  0 '***' 0.001 '**' 0.01 '*' 0.05 '.' 0.1 ' ' 1
```

```r
anova(M2,M3,test="Chisq")
```

```
## Analysis of Deviance Table
##
## Model 1: Dist.m ~ Date + TechP
## Model 2: Dist.m ~ TechP
##   Resid. Df Resid. Dev Df Deviance  Pr(>Chi)
## 1       202     736461
## 2       203     800506 -1   -64045 < 2.2e-16 ***
## ---
## Signif. codes:  0 '***' 0.001 '**' 0.01 '*' 0.05 '.' 0.1 ' ' 1
```

```r
anova(M2,M4,test="Chisq")
```

```
## Analysis of Deviance Table
##
## Model 1: Dist.m ~ Date + TechP
## Model 2: Dist.m ~ Date
##   Resid. Df Resid. Dev Df Deviance  Pr(>Chi)
## 1       202     736461
## 2       205     873380 -3  -136919 < 2.2e-16 ***
## ---
## Signif. codes:  0 '***' 0.001 '**' 0.01 '*' 0.05 '.' 0.1 ' ' 1
```

##2.4 GLM model diagnosis

```r
par(mfrow=c(2,2))
plot(M1)
```

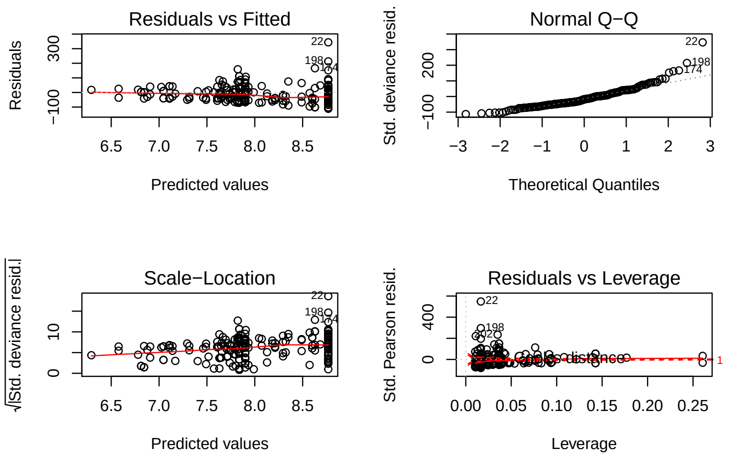

There are some outliers - those are individuals who have boats with engine, and so they go much further from the shore. The results are similar when the models are run without them. I Chose to keep the outliers in because it is an adaptation and it does not affect model fitting.

2.5 GLM plotting

```
Fi <- Fi[complete.cases(Fi$Dist.m),]
Fi <- Fi[complete.cases(Fi$TechP),]

int <- M1$coefficients[1] #baseline; intercept for year=0 and technique=Hook
Dat <- M1$coefficients[2] #multiplicative term for one unit increase in time
Net <- M1$coefficients[3] #relative to hook
Div <- M1$coefficients[4]
Trp <- M1$coefficients[5]
Dat_Net <- M1$coefficients[6]
Dat_Div <- M1$coefficients[7]
Dat_Trp <- M1$coefficients[8]

ggplot(data=Fi,aes(x=Date,y=Dist.m)) +
  geom_point(shape=21,colour="black",size=3,aes(fill=TechP))+
  stat_function(colour="navyblue",size=2, fun=function(Date) exp(int+Dat*Date))+
  stat_function(colour="red",size=2, fun=function(Date) exp(int+Net+(Dat+Dat_Net)*Date ))+
  stat_function(colour="seagreen3",size=2, fun=function(Date) exp(int+Div+(Dat+Dat_Div)*Date )) +
  stat_function(colour="gold",size=2, fun=function(Date) exp(int+Trp +(Dat+Dat_Trp ) *Date))+
  scale_fill_manual(values=c("navyblue" ,"red" ,"seagreen3" ,"gold"))+
  xlab("Year") + ylab("Distance (m)") +
  theme_classic()+
  ylim(c(0,15000))+
  theme(axis.text=element_text(size=16,face="bold"),
        axis.title=element_text(size=22,face="bold"),
        panel.grid.major.y = element_blank(),
        panel.grid.major.x = element_line(colour = "grey"),
        legend.title=element_blank(),
        legend.text=element_text(size=14))
```

```
## Warning: Removed 4 rows containing missing values (geom_point).
```

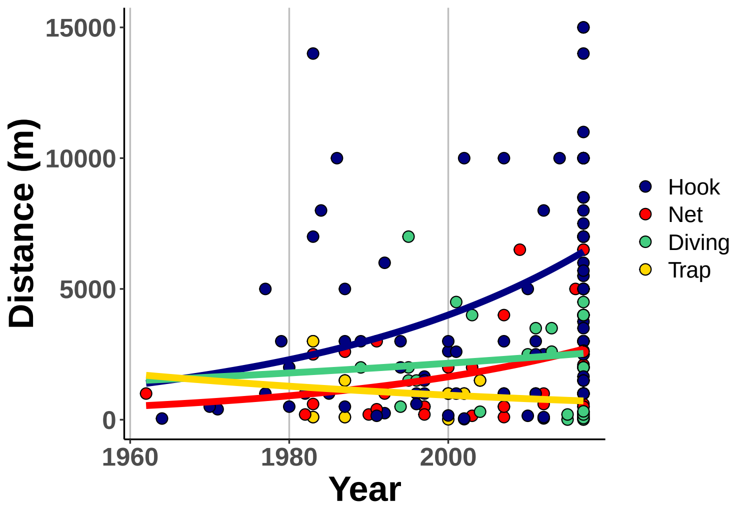

15000
10000
5000
0
Distance (m)
1960
1980
2000
Year
Hook
Net
Diving
Trap
