# Supplementary material for: What can be learned from fishers’ perceptions for fishery management planning? Case study insights from Sainte-Marie, Madagascar
Source: PLoS One. 2021 Nov 15;16(11):e0259792. doi: 10.1371/journal.pone.0259792 (PMC8592436; doi:10.1371/journal.pone.0259792)

Supplementary file S2: R scripts for Multiple Factor Analysis

Written by: Thaïs Bernos

We used Multiple Factor Analysis (MFA) to explore how individual's propensity to suggest management restrictions was associated with individual characteristics, site-level characteristics, coping mechanisms, and perceptions related to ecological health. Tailored to accommodate qualitative variables, MFA is an extension of Principal Component Analysis in which variables within the same groups are weighted to balance the importance of the groups.

To run it, you will need the following packages;

```
library(FactoMineR)
library(factoextra)
library(ggbiplot)
library(psych)
library(paran)
library(ggplot2)
library(gridExtra)
```

1. Load data

For this analysis, we will use the SM_MFA.txt dataset. In the code below, I named it Mada_indexes.

```
summary(Mada_indexes)
```

```
##      Tourism        Leisure        Shops        Lagoons        Gender
##  LA_hot   :54   LA_leis   :50   LA_sho   :68   LA_lag   :81   IN_man   :101
##  LA_hotno:63   LA_leisno:67   LA_shono:49   LA_lagno:36   IN_woman: 16
##
##       Age        Association    Income.div    Attachment   Score_FishDist
##  IN_old   :60   IN_Asso   :36   IN_dep   :59   IN_att   :84   ED_dist   :54
##  IN_young:57   IN_Assono:81   IN_depno:58   IN_Attno:33   ED_distno:63
##
##    Score_FishAb   Score_FishDisp   Score_FishSiz       Score_CopR
##  ED_Num   :110   ED_disp   :55     ED_Size   :58    CR_adapt    :75
##  ED_Numno:   7   ED_dispno:62     ED_sizeno:59    CR_continue:17
##                                                   CR_decrease:25
##      Score_CopingH     Score_Causes Score_Solution
##  CH_adapt    :26    Co_fishing:98    RE_no :44
##  CH_continue:23    Co_notfish:19    RE_yes:73
##  CH_decrease:68
```

All variables are categorical in this analysis. Note that you could use a mix of continuous and categorical variables but, within groups, all variables have to be the same type. Variables were coded as described in Table 1 prior to running the MFA.

Prior to running the MFA, we ran the Keyser-Meyer-Olkin (KMO) and Bartlett's test of homogeneity of variances implemented in "psych" to confirm sampling adequacy.

```
#Make data frames
mydf<- data.matrix(Mada_indexes)

#KMO test; MSA >0.5 indicates adequate sampling.
KMO(mydf) # KMO between 0.6-0.7; This shows that we have adequate sampling
```

```
## Kaiser-Meyer-Olkin factor adequacy
## Call: KMO(r = mydf)
## Overall MSA =  0.61
## MSA for each item =
##        Tourism        Leisure          Shops        Lagoons         Gender
##           0.61           0.67           0.56           0.59           0.65
##            Age    Association     Income.div     Attachment Score_FishDist
##           0.47           0.61           0.46           0.57           0.72
##   Score_FishAb Score_FishDisp  Score_FishSiz     Score_CopR  Score_CopingH
##           0.37           0.68           0.60           0.57           0.46
##   Score_Causes Score_Solution
##           0.76           0.80
```

```
#Bartlett est; p<0.05 indicates adequate sampling
cortest.bartlett(mydf) # p<0.05; this indicates that we have adequate sampling.
```

```
## R was not square, finding R from data
```

```
## $chisq
## [1] 427.4267
##
## $p.value
## [1] 7.661035e-32
##
## $df
## [1] 136
```

2. Run MFA

First, use Horn's parallel analysis to determine the number of dimensions retained in the MFA.

```
#First, check how many dimensions should be retained.
paran(mydf,graph=FALSE)
```

```
##
## Using eigendecomposition of correlation matrix.
## Computing: 10%  20%  30%  40%  50%  60%  70%  80%  90%  100%
##
##
## Results of Horn's Parallel Analysis for component retention
## 510 iterations, using the mean estimate
##
## --------------------------------------------------
## Component   Adjusted    Unadjusted    Estimated
##             Eigenvalue  Eigenvalue    Bias
## --------------------------------------------------
## 1           2.260890    2.994278      0.733387
## 2           1.583344    2.156860      0.573515
## 3           1.105483    1.557441      0.451958
## 4           1.068277    1.413882      0.345604
## 5           1.102063    1.358996      0.256932
## --------------------------------------------------
##
## Adjusted eigenvalues > 1 indicate dimensions to retain.
## (5 components retained)
```

Now, run MFA. According the Horn's analysis, we are only keeping the first 5 dimensions. Support for restrictions is specified as a supplementary variable.

```
MFA_All  <-  MFA(Mada_indexes,
                 group=c(4,5,4,2,1,1), #defines the group of variables
                 type=rep("n",6), # n for categorical groups
                 ncp=5,#indicates that 5 dimensions should be retained
                 name.group=c("Assets","Individual","Eco health","Coping", "Causes","Solution"),
                 num.group.sup =6,# this allows you to interpret the link with the axis
                 graph=FALSE)

# proportion of the variance explained by dimension 1-4
print(MFA_All$eig[1:5,])
```

```
##        eigenvalue percentage of variance cumulative percentage of variance
## comp 1  2.0806803              17.054047                          17.05405
## comp 2  1.4221133              11.656182                          28.71023
## comp 3  1.1180642               9.164080                          37.87431
## comp 4  1.0205363               8.364704                          46.23901
## comp 5  0.8832984               7.239850                          53.47886
```

Make scree plot to visualize the proportion of variance explained by each dimension (we are only keeping the first five).

```
fviz_screeplot(MFA_All, addlabels = TRUE, ylim = c(0, 25))+
    theme(axis.text=element_text(size=20,face="bold"),axis.title=element_text(size=20,face="bold"),
        text=element_text(size=20),
        panel.grid.major.y = element_line(colour = "grey95"),
        panel.grid.major.x = element_line(colour = "grey95"),
        panel.border = element_rect(colour = "black", fill=NA, size=2),
        plot.title = element_text(hjust = 0.5))
```

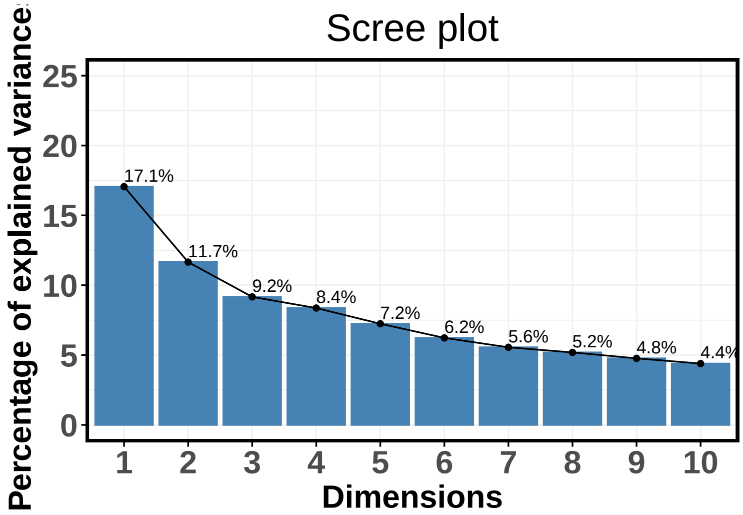

3. Interpret MFA

To interpret the output of the MFA, we followed the following steps:

- We used the v.test statistic to identify significant association (v.test >2) between support for restrictions and the dimensions

- We identified the variables that were the most significantly associated (R2>0.20, p<0.05) with each dimension to describe the data

```
# proportion of the variance explained by the groups
MFA_All$group$contrib
```

```
##                 Dim.1     Dim.2     Dim.3     Dim.4     Dim.5
## Assets      3.482605 32.628973  5.080510  4.626312 42.088273
## Individual 26.302720 29.494989 26.656856 27.582900 36.236636
## Eco health 25.317408 13.029895 44.905282  7.416686 10.285637
## Coping     26.346831 17.501900 13.782429 58.199569  5.423964
## Causes     18.550436  7.344243  9.574922  2.174534  5.965490
```

```
MFA_All$quali.var.sup$v.test #only significant for dimension 2
```

```
##           Dim.1     Dim.2      Dim.3      Dim.4      Dim.5
## RE_no   4.82012 -1.434211 -0.8268101  0.8592316 -0.6925396
## RE_yes -4.82012  1.434211  0.8268101 -0.8592316  0.6925396
```

```
#R2 for dimension 1 (can be computed for other dimensions) - only outputting the first few lines of dimension one b
dim_one <- dimdesc(MFA_All,axes=1,proba=0.05)
head(dim_one$Dim.1$quali) # look at the variables that have an R2 > 0.2
```

```
##                        R2      p.value
## Gender          0.5177172 6.310457e-20
## Score_Causes    0.3859753 7.804312e-14
## Score_FishDisp  0.3386953 5.913755e-12
## Score_CopingH   0.3605349 8.542000e-12
## Score_CopR      0.3568500 1.185217e-11
## Score_FishDist  0.2906229 3.595683e-10
```

```
head(dim_one$Dim.1$category) # interpret the categories accordingly.
```

```
##                             Estimate      p.value
## Gender=IN_woman            1.5103728 6.310457e-20
## Score_Causes=Co_notfish    1.2149221 7.804312e-14
## Score_FishDisp=ED_dispno   0.8409805 5.913755e-12
## Score_CopingH=CH_continue 1.4713576 5.117901e-11
## Score_FishDist=ED_distno   0.7799312 3.595683e-10
## Score_Solution=RE_no       0.6663458 4.214179e-07
```

4. Make Plots

Dimension one is the only one that is statistically significantly associated with our main variable of interest (propensity to suggest management restrictions). For this reason, I am only looking at dimension 1 and 2. Since we included support for restrictions as a supplementary variable: individuals that are close to one another in multidimensional space share similar individual attributes, perceptions of ecological health, underlying causes, coping mechanisms, or local assets.

```
Varplot <- fviz_mfa_var(MFA_All, "quali.var",repel=TRUE,labelsize=3,col.var="black",title="")
coord <- as.data.frame(MFA_All$quali.var.sup$coord)

fviz_add(Varplot, coord, color ="red")+
  coord_fixed()+
  theme(axis.text=element_text(size=20,face="bold"),axis.title=element_text(size=20,face="bold"),
        text=element_text(size=20),
        panel.grid.major.y = element_line(colour = "grey95"),
        panel.grid.major.x = element_line(colour = "grey95"),
        panel.border = element_rect(colour = "black", fill=NA, size=2),
        plot.title = element_text(hjust = 0.5))
```

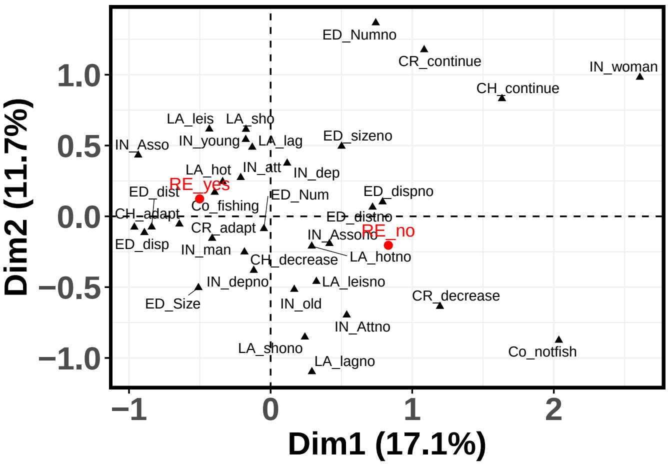


```
fviz_mfa_ind(MFA_All,label="none",habillage=Mada_indexes$Score_Solution,pointsize=4,addEllipses=TRUE,title="") +
                coord_fixed()+
                theme(axis.text=element_text(size=20,face="bold"),
                      axis.title=element_text(size=20,face="bold"),text=element_text(size=2),
                      panel.grid.major.y = element_line(colour = "grey95"),
                      panel.grid.major.x = element_line(colour = "grey95"),
                      panel.border = element_rect(colour = "black", fill=NA, size=2),
                      plot.title = element_text(hjust = 0.5))
```

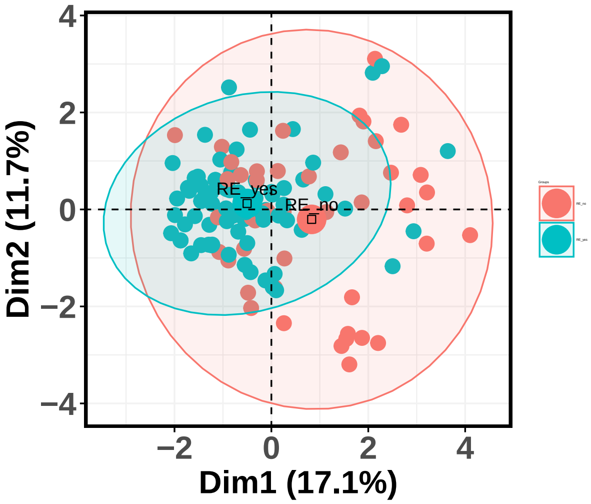

Supplement: S2 File — (PDF) [file pone.0259792.s016.pdf]
