# Supplementary material for: What can be learned from fishers’ perceptions for fishery management planning? Case study insights from Sainte-Marie, Madagascar
Source: PLoS One. 2021 Nov 15;16(11):e0259792. doi: 10.1371/journal.pone.0259792 (PMC8592436; doi:10.1371/journal.pone.0259792)

PLOS ONE

What can be learned from fishers' perceptions for fishery management planning?

--Manuscript Draft--

**Manuscript Number:**	PONE-D-21-12968
**Article Type:**	Research Article
**Full Title:**	What can be learned from fishers' perceptions for fishery management planning?
**Short Title:**	Examining fisher’s perceptions to inform fishery management planning
**Corresponding Author:**	Thais Bernos<br>GRET Professionals for Fair Development<br>Soavimbahoaka, MADAGASCAR
**Keywords:**	conservation social science; small-scale fishery; Perception; adaptive capacity; Madagascar
**Abstract:**	Local support is critical to the success of fishery management initiatives and their longevity. How resource users perceive ecological changes, explain them, and cope with them is strongly related to local support. Here we focused on the small-scale fishery of Sainte-Marie Island, in Madagascar. We assessed perceptions both qualitatively and with composite indexes; we also established a knowledge baseline of the fishery. Our goal was to inform the early stages of fishery management planning. We found that Sainte-Marie’s fishery bear the hallmarks of a transitioning socio-ecological system with very diverse, and strongly gendered fishery practices. Fishers unanimously perceive that the fishery resources are degraded; however, perceptions related to the underlying causes and potential solutions to the observed changes were more heterogeneous among fishers and geographical locations. Similarly, fishers’ coping mechanisms were variable, and realized coping mechanisms (responses to the perceived degradation of the fishery) were often in sharp contrast to the stated willingness to exit the fishery if declines persisted. We suggest that greater attention to good governance, fishers’ expectations from management outcomes, and the local factors influencing fishers’ responses to fishery changes, might help ensure that the initiative will be successful. Collectively, our study of Sainte-Marie’s fishery highlights that dialogues with local fishers is essential to ensure that fishery management plans are adapted to the local needs and context.
**Order of Authors:**	Thais Bernos
	Clodio Travouck
	Naly Ramasinoro
	Dylan Fraser
	Barbara Mathevon
**Additional Information:**	
**Question**	**Response**
**Financial Disclosure**<br><br>Enter a financial disclosure statement that describes the sources of funding for the work included in this submission. Review the submission guidelines for detailed requirements. View published research articles from *PLOS ONE* for specific examples.<br><br>This statement is required for submission and **will appear in the published article** if	The author(s) received no specific funding for this work.

the submission is accepted. Please make sure it is accurate.<br><br>**Unfunded studies**<br>Enter: *The author(s) received no specific funding for this work.*<br><br>**Funded studies**<br>Enter a statement with the following details:<br>• Initials of the authors who received each award<br>• Grant numbers awarded to each author<br>• The full name of each funder<br>• URL of each funder website<br>• Did the sponsors or funders play any role in the study design, data collection and analysis, decision to publish, or preparation of the manuscript?<br>• **NO** - Include this sentence at the end of your statement: *The funders had no role in study design, data collection and analysis, decision to publish, or preparation of the manuscript.*<br>• **YES** - Specify the role(s) played.<br><br>* typeset	
**Competing Interests**<br><br>Use the instructions below to enter a competing interest statement for this submission. On behalf of all authors, disclose any competing interests that could be perceived to bias this work—acknowledging all financial support and any other relevant financial or non-financial competing interests.<br><br>This statement is required for submission and **will appear in the published article** if the submission is accepted. Please make sure it is accurate and that any funding sources listed in your Funding Information later in the submission form are also declared in your Financial Disclosure statement.<br><br>View published research articles from *PLOS ONE* for specific examples.	The authors have declared that no competing interests exist.

**NO authors have competing interests**<br><br>Enter: *The authors have declared that no competing interests exist.*<br><br>**Authors with competing interests**<br><br>Enter competing interest details beginning with this statement:<br><br>*I have read the journal's policy and the authors of this manuscript have the following competing interests: [insert competing interests here]*<br><br>* typeset	
**Ethics Statement**<br><br>Enter an ethics statement for this submission. This statement is required if the study involved:<br><br>• Human participants<br>• Human specimens or tissue<br>• Vertebrate animals or cephalopods<br>• Vertebrate embryos or tissues<br>• Field research<br><br>Write "N/A" if the submission does not require an ethics statement.<br><br>General guidance is provided below. Consult the submission guidelines for detailed instructions. **Make sure that all information entered here is included in the Methods section of the manuscript.**	All the activities carried out by the authors at Sainte-Marie (including this study) were reviewed and approved by the decentralized technical services of the State and local authorities prior to being conducted. This study was exempt from ethics board approval because it results from the secondary use of information collected as part of service-oriented activities, and the data presented is anonymous and aggregated. There is no human research ethics committee overseeing the activities of non-governmental associations in Madagascar. However, we followed published recommendations for ethical research conduct in Madagascar (1): for instance, we adhered to common principles of human research ethics, sought approval from local authorities and respondents, and shared outcomes with local stakeholders.<br><br>1. Wilmé L, Waeber P, Moutou F, Gardner C, Razafindratsima O, Sparks J, et al. A proposal for ethical research conduct in Madagascar. Madag Conserv Dev. 2016;11(1):36–9.

Powered by Editorial Manager® and ProduXion Manager® from Aries Systems Corporation

**Format for specific study types**<br><br>**Human Subject Research (involving human participants and/or tissue)**<br>• Give the name of the institutional review board or ethics committee that approved the study<br>• Include the approval number and/or a statement indicating approval of this research<br>• Indicate the form of consent obtained (written/oral) or the reason that consent was not obtained (e.g. the data were analyzed anonymously)<br><br>**Animal Research (involving vertebrate animals, embryos or tissues)**<br>• Provide the name of the Institutional Animal Care and Use Committee (IACUC) or other relevant ethics board that reviewed the study protocol, and indicate whether they approved this research or granted a formal waiver of ethical approval<br>• Include an approval number if one was obtained<br>• If the study involved *non-human primates*, add *additional details* about animal welfare and steps taken to ameliorate suffering<br>• If anesthesia, euthanasia, or any kind of animal sacrifice is part of the study, include briefly which substances and/or methods were applied<br><br>**Field Research**<br><br>Include the following details if this study involves the collection of plant, animal, or other materials from a natural setting:<br>• Field permit number<br>• Name of the institution or relevant body that granted permission	
**Data Availability**<br><br>Authors are required to make all data underlying the findings described fully available, without restriction, and from the time of publication. PLOS allows rare exceptions to address legal and ethical concerns. See the PLOS Data Policy and FAQ for detailed information.	No - some restrictions will apply

A Data Availability Statement describing where the data can be found is required at submission. Your answers to this question constitute the Data Availability Statement and **will be published in the article**, if accepted.<br><br>**Important:** Stating 'data available on request from the author' is not sufficient. If your data are only available upon request, select 'No' for the first question and explain your exceptional situation in the text box.<br><br>Do the authors confirm that all data underlying the findings described in their manuscript are fully available without restriction?	
**Describe where the data may be found in full sentences. If you are copying our sample text, replace any instances of XXX with the appropriate details.**<br><br>• If the data are **held or will be held in a public repository**, include URLs, accession numbers or DOIs. If this information will only be available after acceptance, indicate this by ticking the box below. For example: *All XXX files are available from the XXX database (accession number(s) XXX, XXX.).*<br>• If the data are all contained **within the manuscript and/or Supporting Information files**, enter the following: *All relevant data are within the manuscript and its Supporting Information files.*<br>• If neither of these applies but you are able to provide **details of access elsewhere**, with or without limitations, please do so. For example:<br><br>*Data cannot be shared publicly because of [XXX]. Data are available from the XXX Institutional Data Access / Ethics Committee (contact via XXX) for researchers who meet the criteria for access to confidential data.*<br><br>*The data underlying the results presented in the study are available from (include the name of the third party*	Data will be held in a public repository.

and contact information or URL).<br>• This text is appropriate if the data are owned by a third party and authors do not have permission to share the data.<br><br>* typeset	
Additional data availability information:	Tick here if the URLs/accession numbers/DOIs will be available only after acceptance of the manuscript for publication so that we can ensure their inclusion before publication.

Powered by Editorial Manager® and ProduXion Manager® from Aries Systems Corporation

Title plage Click here to access/download;Manuscript;Title page.docx

What can be learned from fishers' perceptions for fishery management planning? Case study insights from Sainte-Marie, Madagascar

Fisher's perceptions to inform fishery management planning

T.A. Bernos[1*], C. Travouck[1], N. Ramasinoro[1], D.J. Fraser[2], B. Mathevon[3]

[1] Natural resources management program, Gret Professionals for Fair Development, Soavimbahoaka, Madagascar

[2] Department of Biology, Concordia University, Montreal, Quebec, Canada

[3] Natural resources management program, Gret Professionals for Fair Development, Nogent sur Marne cedex, France

*Corresponding author now at: Department of Biological Sciences, University of Toronto Scarborough, 1265 Military Trail, M1C1A4 Toronto, Ontario, Canada.

E-mail addresses: thais@bernos.fr (T.A. Bernos)

Manuscript Click here to access/download;Manuscript;SainteMarie.docx

Abstract

Local support is critical to the success of fishery management initiatives and their longevity. How resource users perceive ecological changes, explain them, and cope with them is strongly related to local support. Here we focused on the small-scale fishery of Sainte-Marie Island, in Madagascar. We assessed perceptions both qualitatively and with composite indexes; we also established a knowledge baseline of the fishery. Our goal was to inform the early stages of fishery management planning. We found that Sainte-Marie's fishery bear the hallmarks of a transitioning socio-ecological system with very diverse, and strongly gendered fishery practices. Fishers unanimously perceive that the fishery resources are degraded; however, perceptions related to the underlying causes and potential solutions to the observed changes were more heterogeneous among fishers and geographical locations. Similarly, fishers' coping mechanisms were variable, and realized coping mechanisms (responses to the perceived degradation of the fishery) were often in sharp contrast to the stated willingness to exit the fishery if declines persisted. We suggest that greater attention to good governance, fishers' expectations from management outcomes, and the local factors influencing fishers' responses to fishery changes, might help ensure that the initiative will be successful. Collectively, our study of Sainte-Marie's fishery highlights that dialogues with local fishers is essential to ensure that fishery management plans are adapted to the local needs and context.

Keywords: conservation social science, small-scale fishery, perception, adaptive capacity, Madagascar

1. Introduction

Many coastal communities depend on marine resources for their livelihood and well-being. Ensuring that local needs for marine resources are met without compromising those of future generations often requires the development of fishery management plans –sets of rules guiding the conservation and sustainable use of marine resources. The short-term effectiveness and long-term persistence of those fishery management plans are heavily influenced by local community support (1,2). Support for fishery management is in part determined by resource users ability to adapt and implement management actions, which is in turn influenced by their dependence on marine resources, access to livelihood alternatives, demographic, and socioeconomic status (3–5). It is also influenced by perceptions - how individuals observe, interpret, or evaluate their environment- related to fshery management (5–8).

In small-scale fisheries, resource user perceptions about the current state of the fisheries, preferred ways to address existing issues, and coping strategies, strongly influence levels of support for fishery management (5,9,10). In particular, resource users may be more supportive of fishery management if they: i) believe it would benefit themselves and their community via ecosystem services (11), ii) agree on the type of management they would prefer to implement (12), and iii) feel that they can adapt to those changes (13). Of course, perceptions tend to vary greatly between individuals and communities as they are shaped by a mosaic of factors, including the extent of an individual's local ecological knowledge, their activities in the fishery, level of education, and position in existing social networks (3,14,15). Understanding the heterogeneity in perception between resource users is beneficial for fishery management as it can help uncover opportunities or potential challenges for fishery management (16–18).

On the island nation of Madagascar, fishing is critically important for the economy, nutritional health, and food security of a large number of coastal communities (19). Many fisheries, however, are dwindling (20,21). As fishery use is essentially uncontrolled, marine resources should benefit from management restrictions (20). Substantial efforts have been made to manage marine resources more sustainably, including the creation of a network of coastal water areas co-managed locally by local stakeholders, referred to as Locally Managed Marine Areas (LMMAs) (22). Those initiatives typically really heavily on international financial support, national legislation, local governance, and local enforcement (23,24). In a context of limited state-capacity to enforce legislation, fishery rules are often expected to be self or community-enforced.

Here, our objective was to evaluate fishers' perceptions and their ability to adapt to changes in the fishery. We focus on an island off northeastern Madagascar, Sainte-Marie (*Nosy boraha*: Figure 1), on which future fishery management plans were extensively discussed at community meetings when the present study was conducted. Specifically, we i) evaluate fishers perceptions related to the ecological health of the fishery, current issues and potential solutions, and coping strategies; ii) contrast heterogeneity in perceptions between individuals and geographic locations. Prior to conducting this study, Sainte-Marie's fishery had not been studied: we therefore

lacked a basic understanding of fishery practices, as well as a knowledge baseline of the dynamics of the fishery. Consequently, we also iii) characterize the diversity of fishing practices and iv) reconstruct temporal trends in fishery dynamics as seen from fishers' perspectives. Our intent was to help design a fishery management plan reflecting the local context and fishers' preferences.

2. Study area and context

Sainte-Marie is inhabited by 25-30,000 people; while the exact number of fishers is unknown, locals establish that "everyone fishes". We studied 16 of the 17 *fokontany* (villages), arranged in four boroughs. We could not find any fishers in Ambohitry, the only village that is not located directly on the shorelines. These villages present several ecological and socioeconomic differences. Notably, infrastructures (e.g., hotels, airport, port, seafood processing factory) are concentrated in the southern boroughs, providing more access to alternate livelihoods. Nonetheless, most people live below the poverty line. The western boroughs are separated from Madagascar's mainland by the 7-23km wide Sainte-Marie canal; the eastern boroughs are exposed to the ocean. The Southeastern borough harbours most of the near-shore coral reefs and lagoons. The humid tropical climate is favorable to cash crops and trees (i.e. clove trees, vanilla). Extreme weather events from December-March (heavy rainfalls, annual cyclones) affect local livelihoods.

Fishing practices and targets are extremely diversified. Many fishers use non-motorized pirogues, and the main fishing techniques are line-fishing, netting (gill nets, beach seine), free diving, and fish traps. Fishers typically use several fishing techniques: technique use is influenced (among other) by fishers conditions, preferences, as well as target species, weather conditions, and fishing locations. For instance, older fishers are more likely to use fish traps while many younger fishermen free dive; under bad weather conditions, fishers are more likely to be found netting in the lagoons that line fishing further from the shore. Some species, such as mackerels, are often targeted at night using kerosene lamp and line fishing, and shrimps can be targeted with specific nets in the mangroves. Reflecting the wide variety of fishing techniques, Sainte-Marie's fishers target a highly diverse portfolio of species; more than 50 local Malagasy species names were provided (see Table S1 for the species caught the most frequently) including

both mobile (e.g. migratory fishes) and sedentary species (e.g. mollusks, arthropods). As for market, fishers mainly sell their catches to a commercial fishery with an international export market, hotels, restaurants, and locals. In most villages, sous-collectors working for the exporting fishery can store the fishery resources, which are then collected, processed, and packaged for export on the island. As many other small-scale fisheries, fishery participation is very gendered (25–27); women tend to fish for subsistence, target species of lower economic value, and are generally restricted to easily accessible near-shore areas.

Sainte-Marie's nearshore fishery is managed under national fishery laws, but those are not fully complied with and enforced as responsible authorities lack the capacity to enforce them in the area. It is worth noting, however, that a protected area was established in 2014 by the Indian Ocean Commission and some local stakeholders. It generated significant hostility, particularly from the fishers it impacted in the Southern borough (Vohilava). Eventually, the resistance of the communities led to conflicts and the resulting failure of the initiative that same year. Other than western approaches to management, local cultural taboos (*fady*) may also play a role in regulating fishery-related activities. Some *fady* entail species-specific protection: killing Guitarfish is taboo for all, and eating sea turtles for certain families. Other taboos are location specific. For instance, on the sandy islets off the southeastern end of the island, harvesting several types of marine resources (e.g. mollusk and fish) at once, fishing with nets, and polluting the area, are prohibited. Access is also restricted (prohibited to Merina, a highlander ethnic group). When taboos are violated, spiritual sanctions involve mischance, disease, or death; material sanction can also be ordered by the community. However, fishers expressed that taboo enforcement and respect had declined because of several external changes, including the apparition of a new market for guitarfish fins, market multiplication, and harder times. In conclusion, marine resources were essentially open access.

In 2015, the non-governmental organization GRET -Professionels du développement solidaire (professionals for fair development) started working with the communities to improve livelihood sustainability. This led to the creation of the PCADDISM -Plateforme de concertation des acteurs pour le developpement durable de l'ile Sainte-Marie (Concertation platform for sustainable development actors on Sainte Marie Island), a platform managed and led by the communities where local stakeholders (e.g. local fishers' federation, commercial fishery, private hotels) are

represented. The GRET provides technical and financial support to the PCADISM. Inspired by the nation-wide expansion of the LMMA network, the idea to create LMMAs governed by traditional laws (*dina*) had started to gain momentum within the PCADISM. When the present research was conducted, the PCADISM was holding meetings in each village to discuss livelihood sustainability, including possible fishery management strategies. Those meetings were open to all who wished to come, were highly deliberative, and widely attended by villagers.

3. Material and methods

All the activities carried out by the GRET at Sainte-Marie (including this study) were reviewed and approved by the decentralized technical services of the State and local authorities prior to being conducted. This study was exempt from ethics board approval because it results from the secondary use of information collected as part of service-oriented activities, and the data presented is anonymous and aggregated. There is no human research ethics committee overseeing the activities of non-governmental associations in Madagascar. However, we followed published recommendations for ethical research conduct in Madagascar (28): for instance, we adhered to common principles of human research ethics, sought approval from local authorities, and shared outcomes with local stakeholders.

3.1. Survey strategy and design

We surveyed fishers' conditions and perceptions between June-October 2017 (Table S2: Table S3 for sample sizes per village). Interviewees were selected based on randomized and peer-referenced sampling. Informants spanned a wide range of ages (18-71 years old) and represented fishing technique use in each of the villages. Upon arrival in a village, we met with traditional authorities, informed them about the purpose and intended use of the survey, and obtained their consent. The interviews were semi-structured and conducted in Malagasy. Interviews lasted between 30min and 3 hours. Before conducting the survey, fishers were told about the purpose of the survey and its intended use, and we obtained verbal consent.

This paper focuses on fishers' characteristics and their perceptions related to the current state of the fishery, preferred ways to address existing issues, and coping strategies (Table 1 and Table S4). For each of those topics, we focused on multiple items to build an index. Items were selected based on theoretical or previously demonstrated relationships with fishery livelihoods and capacity to adapt to fishery changes in similar contexts. Perceptions related to the ecological state of the fishery were evaluated with questions related to perceived changes in fish abundance, distance to fishing site, local extinction, and fish sizes (29,30). To evaluate existing issues and preferred ways to address them, fishers were asked to identify the causes of the perceived changes as well as potential solutions (12,31). Finally, to investigate coping strategies, we asked fishers how they had responded to observed changes in the fishery and how they would respond to an hypothetical scenario of further decline (32,33).

To build a knowledge baseline regarding past changes in the fishery, we used the methodology described in other studies eliciting fishers' memories (e.g. Tesfamichael et al. 2014; Damasio et al. 2015; Bevilacqua et al. 2016; Melo et al. 2015). Specifically, fishers were asked to remember what a great catch would be for the main species they caught for two given years (2017, the year we conducted this study; and 2009, the year a major cyclone hit the island) as well as the year they started fishing. We focused on "great catches" as, consistently with other empirical (Tesfamichael et al. 2014; Melo et al. 2015) and cognitive studies (Bradburn 1987; Rubin and Kozin 1984), asking fishers about an average catch size did not elicit precise answers, but they were able to estimate great catches. Oftentimes, people did not know catch size with precision but knew how many fish boxes they landed and the approximate number of corresponding kilograms. We specifically chose 2009 as it was more likely to be anchored in memories since a cyclone caused substantial damages on Sainte-Marie that year. Data validity was verified at different times during the research. For instance, during the interview, answers were queried for unrealistic answers. Interviewers were also able to cross-check the provided weight.

3.2. Ethical considerations

3.3. Analysis

First, we used descriptive tables to summarize fishers' characteristics and evaluate their perceptions related to the current state of the fishery, preferred ways to address existing issues, and coping strategies. Prior to building the tables, responses to the qualitative questions were organized into categories which we defined after all responses had been read. Due to marked differences between men's and women's socioeconomic characteristics, activities in the fishery, and perceptions, we discuss results separately between genders.

Second, we evaluate heterogeneity in perceptions between individuals and geographic locations. To do so, we computed individual perception indexes by combining items related to, respectively, state of the fishery, current issues and solutions, and coping strategies. Individual perceptions indexes were only computed for individuals for which all items could be scored (n=104), all of which were men. We excluded two fishers who seasonally traveled to remote mainland locations for fishing as their coping strategies (realized and hypothetical) were external to Sainte-Marie; we also excluded two fishers with incomplete responses. To do so, we scored items related to each topic based on previously demonstrated relationships with support for management (See Table S4). To compute the composite indexes for each topic, we first normalized each of the item score on a scale of 0-2, then summed each normalized item score within each topic group and normalized the final score on a scale of 0-10 to allow comparisons (as in Bennett et al. 2019). We added the three perception indexes to create a total index of perception on a 0-30 scale.

To explore differences in perceptions among geographic locations, we used Kruskal-Wallis tests to test for significant differences between individual perception indexes among boroughs, as well as composite indexes for each of the three topics. Significant Kruskal-Wallis tests were followed by Dunn tests as a post-hoc analysis; we used the package FSA (37), and p-values were adjusted for multiple testing using benjamini-Hochberg FDR adjustment method (38). Last, we explored relationships among perception indexes and respondent characteristics (age, fishing experience, association membership, education, alternative livelihoods) shown to influence

perceptions in similar systems using correlation plots in the corrplot package (39). All statistical analyses were performed in R (40).

Finally, we assessed perceived catch changes over time for 13 commercially-important species identified from the interviews (Table S1). To do so, we used generalized additive mixed modeling (GAMM) (41,42) as preliminary exploration of catch volume over time using linear models suggested poor model fit, a likely consequence of markedly non-linear changes over time. Basal variations among fishermen were accounted for by the inclusion of a fisherman smoother term in the model. A full model was fitted with a species-by-time interaction term to test for differences in timing and extent of catch changes amongst species and catch volume as a response variable. Continuous variables were centered prior to model fitting. Fit was assessed using likelihood ratio tests.

4. Results

4.1. Fishers' characteristics

In total, we interviewed 127 fishers, including 109 men and 18 women (Table S2). There were strong gender-based differences in fishing practices (Table 2). For instance, most fishermen (90%) fished from non-motorized pirogues for commercial species and typically used more than one fishing gear. Among the woman surveyed, most (94%) fished on foot close to their villages: they almost exclusively fished with nets for a diversity of small fishes. Men typically sold their catches to more than one market, of which the villages and the exporting fishery were the most frequently cited (82 and 60%, respectively); by contrast, women predominantly fished to feed their families.

There were also marked socioeconomic differences between men and women. Most of the fishers we surveyed had no formal education (60 vs 72%, respectively men and women) and reported low incomes (122 VS 61 USD$). Men were overall more invested in the fishery; for instance, many men were members of the local fishers association while women were rarely members (64 vs. 6%), and owned expensive fishing gear, such as boats and nets, more regularly (83 vs 39%). Half of the fishermen and few women (51 and 22%) increased their household incomes with other sources of income, typically from agriculture or cash crops.

4.2. Perceptions

Ecological health of the fishery- Overall, respondents painted the picture of a fishery in decline. Both genders almost unanimously (93 and 100% of, respectively, men and women) perceived a general reduction in fish abundance; however, men observed additional changes in the fishery (Table 2). For instance, several fishermen indicated a decrease in fish size (58%) and many (56%) noticed the local disappearance of species. In total, fishers provided 31 local Malagasy names (including 29 fishes) of species that had disappeared from their catches and the marine ecosystems, among which dugongs (*Dugong dugong:* last sighting in the area dates from 1993, Cockroft 1993) and two Mullidae were predominantly listed.

Existing issues and preferred ways to address them- Perceived causes of the decline differed by gender (Table 3). Notably, a much greater proportion of the fishermen attributed it to local fishing compared to women (84 vs. 59%). Causes to the decline related to local fishing agency included increased numbers of fishers (60 and 33%), changes in net-fishing practices (i.e. longer nets, smaller mesh sizes; 34% of men). By contrast, women's most frequently perceived causes for the decline were gods' punishments (44%). Beside local human agency and general causes, it is also important to highlight that several fishers (24 and 11% of men and women) attributed the decline to foreign fishing in Sainte-Marie's waters; they referred to the commercial exploitation of shrimp in the canal (8%) and sea-cucumbers (20 and 11%) on the nearshore area. Surveyed fishers specifically referred to migrant fishers who visit Sainte-Marie seasonally and temporarily; they mentioned that those fishers typically dive for sea cucumbers with diving cylinder and often collect other marine resources, therefore contributing to the decline.

Proposed activities to improve fishery health also differed by gender (Table 3). Compared to men, women were more likely to offer no solutions (18 vs 33%) or propose to appease the gods (8 vs 28%), for instance via spiritual sacrifices and increased respect for local taboos. By contrast, many men suggested solutions that were related to local fishery restrictions (68 vs 17%); in particular, many proposed marine parks (32%) and restrictions on nets (26%). The proportion of men who suggested area-based restrictions varied per borough, and were as follow; 33% in Ambatourao, 10% in Loukintsy, 11% in Ambodiforatra, and 23% in Vohilava.

Coping strategies- There was a sharp contrast between fisher's realized response to fishery changes and coping mechanisms to future hypothetical changes. In response to the decline in fish abundance, realized coping strategies differed between genders. Men's responses were skewed towards increasing fishing efforts: most (72%) indicated that they had increased their fishing effort (time at sea, distance from the shore, gear), some (17%) indicated that they had decreased their fishing effort to develop alternative sources of incomes, and fewer (11%) reported no changes in fishing effort (Table 3). All the women said that their fishing effort had not changed over time. In contrast, when asked about what they would do to cope with further decline in catches, most fishermen and some fisherwomen stated that they would decrease fishing effort (57 vs 33%). Many of the fisherwoman did not know what their coping strategies would be (39%).

4.3. Perception indexes

The perception indexes (Figure 2) for each of the three perception categories, on a scale of 1-10, were as follows; the perceptions about fishery health had a mean of 6.62 (0-10), perceptions about issues and preferred solutions had a mean of 7.69 (0-10), and those related to coping mechanisms had a median of 7.69 (0-10). The total perception index (scale 0-30) had a mean of 17.81 (min-max=5-28.33).

Overall, the Kruskal-Wallis test indicated no significant differences in individual perception indexes between boroughs (χ^2=5.408, p=0.144). There was no difference in the index related to ecological changes in the fishery ($\chi^{2=}$6.222,p=0.10). Nonetheless, there were some significant differences among boroughs in composite indexes related to perceived issues and suggested solutions ($\chi^{2=}$9.5051, p=0.023) and coping mechanisms ($\chi^{2=}$=15.219, p<0.01). When those composite indexes were analyzed separately, Dunn tests revealed that Vohilava fishers (southwestern borough) had, on average, a significantly higher index (p<0.05) related to perceived issues and suggested solutions that those in Loukintsy (northwestern borough). However, Loukintsy fishers had, on average, higher indexes of coping mechanisms than these of the three other boroughs.

We found no significant correlation between the composite indexes of perceptions and socioeconomic variables. However, some of the socioeconomic variables were correlated; in particular, the number of years in the fishery and age were strongly correlated with each other, and negatively correlated with education levels ($p<0.05$). Additionally, our composite index of perceptions related to the state of the fishery was marginally negatively correlated ($0.05<p<0.10$) with perceived issues and suggested solutions.

4.4. Reconstruction of fishery dynamics

According to the fishers, changes in best catch size (highest catch amount) were not linear over time and varied between species (Figure 3, Table S5). The best-fit GAMM included a significant interaction between time and species (LRT=-66181, df=-22.747, $p<0.001$), further implying that perceived catch trajectories differed amongst species over time. Visual plotting confirmed catch decreases for 12 of the 13 species. For 7 species, the decline was gradual since the 1970s; catches increased between 1980 and 2000 for 5 species; catch decline accelerated after 2000 for 1 species. Temporal trends were significant for 4 species ($\alpha \leq 0.05$), marginally significant for 3 ($0.05<\alpha \leq 0.10$), and insignificant for 6 species (Table S5).

5. Discussion

Our analysis of fishers' perceptions helped identify some opportunities and potential challenges for fishery management in Sainte-Marie. Our results mirror those of a growing number of perception studies providing evidence for changes in small-scale fishery catches in Madagascar (31,44–47). Indeed, Sainte-Marie's fishers almost unanimously perceived that marine resources were becoming scarcer. There was no clear difference between boroughs, which indicates that this perception is widespread on the island. Furthermore, there was no significant correlation between perceptions related to the ecological state of the fishery and fishers age, suggesting that even younger generations believe that the abundance and diversity of marine resources are not what they once were. This perception of ecological degradation, shared between many diverse individuals, might provide a strong impetus for fishery management and conservation in Sainte-Marie (5).

Management is more likely to be successful if fishers and their leaders can agree on preferred management restrictions, and work collectively towards their implementation (12). However, perceptions related to causes of fishery changes and preferred solutions were heterogeneous. While many fishers suggested fishery management restrictions, there was no strong consensus about what rules might look like. Some of the restrictions suggested by fishers were unrealistic; for example, it would be unthinkable to ban all fishing from a near-shore area, even for a limited time. Additionally, many felt that nothing could be done due to high dependency on marine resources, or because fisheries were in gods hands. But overall, at such an early stage of fishery management planning, the fact that many fishers perceive local fishing as an important underlying cause to the observed changes and simultaneously suggest fishery management strategies is encouraging.

As fishers continue to discuss fishery management rules, good governance will be key to ensuring the long-term outcomes of fishery management actions. Indeed, when we conducted this study, fishery management options were often discussed at community meetings. Although generally attended by many community members, leadership remained dominated by a select group of powerful individuals. Those were strongly in support of LMMAs: some had been involved in community exchange visits to existing LMMAs, and we suspect that the relative popularity of area restrictions in Sainte-Marie results from local peer exchange following those visits, highlighting the effective model of spread of the nation-wide LMMA network (Mayol 2013; Rocliffe et al. 2014; Riambatosoa Rakotondrazafy 2015). The PCADISM, which is a powerful political platform facilitating community-decision making, is still at its infancy; acting now to facilitate the effective participation of all fishers to the elaboration of fishery management plans and to ensure that a minority of powerful stakeholders do not take power of the initiative might be key to ensure, in the long-term, support for future fishery management actions (48–50).

If fishers decide to move forward with area-based restrictions, the analysis of perception indexes at the borough level provide some insights regarding areas that may be prioritized. Overall, there was no difference in total perception indexes across the entire island. However, when we looked at the three indexes individually, we found

some differences. For instance, Loukintsy had a higher coping mechanism index than the other boroughs. This difference stemmed from the fact that, in response to declining catches, many fishers stated having reduced their fishing effort. However, fishers did not report that they had developed alternative sources of income; rather, they stated that the decline of the fishery had discouraged them from fishing. It is worth mentioning that the fishers we surveyed in Loukintsy were slightly older, on average, than in the other boroughs; and, as there is no lagoon directly facing the borough, the dominant fishing technique is line fishing from pirogues far from the shore. We suspect that the interaction between those two factors might have influenced our result.

There was, however, another element that could provide an opportunity for area-based management. Specifically, we found that Vohilava fishers were more likely to infer human agency as the cause of the decline, and to suggest solutions related to fishery restrictions. Accordingly, support for area-based restriction was relatively high in this borough. Given that Vohilava fishers have a prior negative experience with area-based restrictions, this result was surprising. Some explanations might include: 1) the recognition that fishery restrictions are needed, 2) an awareness of the growth of the nation-wide LMMA network and positive impacts on some communities, 3) greater support for the initiative resulting from a higher engagement of the communities, or 4) a general acquiescence but no real intention to comply with restrictions. Consequently, a greater understanding of the factors affecting fishers' perceptions is needed.

Building realistic expectations for the fishery management plan is important as failing to achieve its objectives would undermine support in the long-term (51). Fishers highlighted important contextual challenges that have proven difficult to address with local fishery co-management initiatives (45,52,53). For instance, seasonal migrants travel to Sainte-Marie (they do not settle on the island) to harvest sea cucumbers, a highly valuable resource on Chinese markets (54). As few local fishers traditionally target sea cucumbers, there should be limited direct competition for those resources. However, there was a strong perception that sea cucumber fishers over-exploit other fishery resources and use illegal fishing techniques. While we did not encounter any sea cucumber fisher migrants in the study, we were shown pictures of SCUBA-equipped diver teams operating in motorized boats (Cetamada, personal communication). This is critical, because a key expectation that local fishers have from fishery

management is that it will help exclude those sea cucumber divers, and to a lesser extend shrimp trawlers, from Sainte-Marie's waters (45,51). Diving with cylinders is currently not permitted, but a lack of capacity for law enforcement and widespread corruption undermines the enforcement of existing rules. While local NGOs could try to promote the sustainable use of sea cucumber by, for instance, helping develop sea cucumber farming (54,55), their capacity to help address those contextual challenges is limited (47,48).

Our research highlights that the relationship between fishers and marine resources had fundamentally changed over the last 50 years. In particular, our reconstruction of fishery changes, which cannot be interpreted independently from larger socioecological transitions, indicates that the fishery gradually transitioned from predominantly subsistence-based to market-based -at least for men. According to discussions with focus groups and individual fishers, people originally fished for subsistence in near-shore areas. In the 1960s, the apparition of new markets, combined with regular boat service established between Sainte-Marie and the mainland, influenced the fishery in several ways; people began to sell their catches, increase fishing efforts, and invest in fishing gear. This explains the increase catch sizes between 1980-2000; notably, the start of the catch growth period for mackerel was congruent with the introduction of kerosene lights used to aggregate them on the island. More recently, however, the voice of Sainte-Marie's fishers join those of other small-scale Malagasy fisher reporting alarming declines (31,44–47). To compensate perceived decline and attempt to maintain their caches, fishers generally increased their fishing efforts; for example, they spent more time at sea, invested in fishing gear, and fished further from the shore (see analysis S1).

As for fishers' stated coping mechanisms to future changes in the fishery, these were in sharp contrast with the aforementioned realized response to fishery changes. Indeed, the most frequent answer to further hypothetical decline was that fishers would pursue alternative livelihoods and reduce fishing effort or exit the fishery. In Sainte-Marie, attachment to fishing may be limited: indeed, fishers perceived fishing as a last resort, risky activity and most (73% and 69%, men and women) ambitioned their children to succeed in education and exit the fishery. As livelihood alternatives are limited on Sainte-Marie, in theory, wealth generation and employment opportunities directed at the resource users could help reduce pressures on natural resources (Ikiara and Odink 2000; McClanahan

et al. 2009). For instance, many fishermen expressed interest (or were already invested) in cash crops as an alternative livelihood. However, other contextual factors might play an opposite role in fisher's capacity and willingness to exit the fishery; in particular, with a well-developed network of sous-collectors in the island, easy access to market could reward fidelity to fishing (32). Consequently, to ensure that fishery management has the intended ecological outcome, understanding the factors affecting fisher's behavior towards exiting the fisheries is vital (4,33,58).

6. Conclusions

Fishery management strategies require the support of local resource users to deliver persisting ecological, social, and economic benefits. Our study of Sainte-Marie's fisheries confirms that dialoguing with local fishers to understand their perceptions is essential to ensure that fishery management plans are adapted to the local needs and context (5,9). Fishers provided a window unto their fisheries, from which the picture of an inherently diverse and temporally dynamic fishery emerged. However, as in other small-scale fishing communities in Madagascar and other island nations, they perceived a widespread decline in the resources that their livelihoods are tightly intertwined with. Despite a challenging context of high reliance on marine resources, a lack of livelihood alternatives, and low state support, many fishers felt that marine ecosystems would benefit from the local establishment of fishery management rules. Moving forward, our results suggest that greater attention to good governance, fishers' expectations from management outcomes, and the local factors influencing fishers' responses to fishery changes, might help ensure that the initiative will be successful.

Ackowledgements

We are infinitely grateful to the fishers of Sainte-Marie for their invaluable support, cooperation, and contributions. We also thank the non-governmental association Cétamada for their technical support. Funding was provided by Agence France Volontaire, Noé Conservation, Find-Gret, and Cétamada. Authors are grateful to the local authorities and traditional chiefs for their invaluable support.

Author contributions

T.A. Bernos designed the study, conducted the interviews and statistical analysis, wrote the manuscript. C. Travouck conducted and translated the interviews. N.R. provided technical support. D.J. Fraser provided writing assistance. B. Mathevon provided theoretical guidance.

Bibliography

1. Bennett NJ, Niccolini F, Franco A Di, Milazzo M, Calò A, Nethery E, et al. Local support for conservation is associated with perceptions of good governance , social impacts , and ecological effectiveness. Conserv Lett. 2019;(December 2018):1–10.

2. Rohe JR, Aswani S, Schlüter A, Ferse SCA. Multiple drivers of local (Non-) compliance in community-based marine resource management : case studies from the South Pacific. Front Mar Sci. 2017;4(172):1–14.

3. Mcclanahan T, Davies J, Maina J, Mcclanahan T, Davies J, Maina J. Factors influencing resource users and managers ' perceptions towards marine protected area management in Kenya Factors. Environ Conserv. 2005;32(1):42–9.

4. Cinner JE, Daw T, Clanahan TRMC. Socioeconomic factors that affect artisanal fishers' readiness to exit a declining fishery. Conserv Biol. 2009;23(1):124–30.

5. Bennett NJ. Using perceptions as evidence to improve conservation and environmental management. Conserv Biol. 2016;30(3):582–92.

6. Jefferson R, Mckinley E, Capstick S, Fletcher S, Grif H, Milanese M. Understanding audiences : Making public perceptions research matter to marine conservation. Ocean Coast Manag. 2015;115:61–70.

7. Gelcich S, O'Keeffe J. Emerging frontiers in perceptions research for aquatic conservation. Aquat Conserv Mar Freshw Ecosyst. 2016;26:986–94.

8. Kittinger JN. Participatory fishing community assessments to support coral reef fisheries comanagement.

Pacific Sci. 2013;67(3):361–81.

9. Beyerl K, Putz O, Breckwoldt A. The Role of Perceptions for Community-Based Marine Resource Management. Front Mar Sci. 2016;3(238).

10. Fabinyi M, Barclay K, Eriksson H. Chinese Trader Perceptions on Sourcing and Consumption of Endangered Seafood. Front Mar Sci. 2017;4(June):1–12.

11. Bennett NJ, Roth R, Klain SC, Chan KMA, Clark DA, Cullman G, et al. Mainstreaming the social sciences in conservation. Conserv Biol. 2016;31(1):56–66.

12. Mcclanahan ATR, Cinner J, Kamukuru AT, Abunge C, Mcclanahan TR, Cinner J, et al. Management preferences , perceived benefits and conflicts among resource users and managers in the Mafia Island Marine Park , Tanzania. Environ Con. 2008;35(4):340–50.

13. Seara T, Clay PM, Colburn LL. Perceived adaptive capacity and natural disasters: A fisheries case study. Glob Environ Chang. 2016;38:49–57.

14. Barnes ML, Lynham J, Kalberg K, Leung P. Social networks and environmental outcomes. PNAS. 2016;113(23):6466–71.

15. Berkes F, Colding J, Folke C. Rediscovery of traditional ecological knowledge as adaptive management. Ecol Appl. 2000;10(5):1251–62.

16. Bennett NJ, Roth R, Klain SC, Chan K, Christie P, Clark DA, et al. Conservation social science : Understanding and integrating human dimensions to improve conservation. Biol Conserv. 2017;205:93–108.

17. Ban NC, Mills M, Tam J, Hicks CC, Klain S, Stoeckl N, et al. A social–ecological approach to conservation planning : embedding social considerations. Front Ecol Environ. 2013;11(4):194–202.

18. Sterling EJ, Betley E, Sigouin A, Gomez A, Toomey A, Cullman G, et al. Assessing the evidence for stakeholder engagement in biodiversity conservation. Biol Conserv. 2017;209:159–71.

19. Barnes-mauthe M, Oleson KLL, Zafindrasilivonona B. The total economic value of small-scale fisheries with a characterization of post-landing trends : An application in Madagascar with global relevance. Fish

Res. 2013;147:175–85.

20. Le Manach F, Gough C, Harris A, Humber F, Harper S, Zeller D. Unreported fishing , hungry people and political turmoil : The recipe for a food security crisis in Madagascar. Mar policy. 2012;36:218–25.

21. Gough CLA, Dewar KM, Godley BJ, Zafindranosy E, Broderick AC. Evidence of overfishing in small-scale fisheries in Madagascar. Front Mar Sci. 2020;7(June):1–17.

22. Mayol TL. Madagascar ' s nascent locally managed marine area network. Madagasvar Conserv Dev. 2013;8(2):91–5.

23. Rakotoson LR, Tanner K. Community-based governance of coastal zone and marine resources in Madagascar. Ocean Coast Manag. 2006;49:855–72.

24. Gardner CJ, Nicoll ME, Mbohoahy T, Oleson KLL, Roland D, Ratsifandrihamanana AN, et al. Protected areas for conservation and poverty alleviation : experiences from Madagascar. J Appl Ecol. 2013;50:1289–94.

25. Nunan F, Cepić D, Mbilingi B, Odongkara K, Yongo E, Owili M, et al. Community Cohesion : Social and Economic Ties in the Personal Networks of Fisherfolk. Soc Nat Resour. 2018;31(3):306–19.

26. Kleiber DL, Harris LM. Gender and small-scale fisheries : A case for counting women and beyond. Fish Fish. 2014;(February).

27. Mcclanahan TR, Abunge C. Fish trader ' s gender and niches in a declining coral reef fishery : implications for sustainability. Ecosyst Heal Sustain. 2017;3(6).

28. Wilmé L, Waeber P, Moutou F, Gardner C, Razafindratsima O, Sparks J, et al. A proposal for ethical research conduct in Madagascar. Madag Conserv Dev. 2016;11(1):36–9.

29. Watson MS, Claar DC, Baum JK. Subsistence in isolation : Fishing dependence and perceptions of change on Kiritimati , the world ' s largest atoll. Ocean Coast Manag. 2016;123:1–8.

30. Leleu K, Alban F, Pelletier D, Charbonnel E, Letourneur Y, Boudouresque CF. Fishers' perceptions as indicators of the performance of Marine Protected Areas (MPAs). Mar Policy. 2012;36(2):414–22.

31. Lemahieu A, Scott L, Malherbe WS, Tsimanaoraty P, Victor J, Aswani S. Local perceptions of environmental changes in fi shing communities of southwest Madagascar. Ocean Coast Manag. 2018;163(May 2017):209–21.

32. Daw TM, Cinner JE, Mcclanahan TR, Brown K, Stead SM, Graham NAJ, et al. To Fish or not to fish : factors at multiple scales affecting artisanal fishers' readiness to exit a declining fishery. PLoS One. 2012;7(2).

33. Cinner JE, Folke C, Daw T, Hicks CC. Responding to change : Using scenarios to understand how socioeconomic factors may influence amplifying or dampening exploitation feedbacks among Tanzanian fishers. Glob Environ Chang. 2011;21(1):7–12.

34. Tesfamichael D, Pitcher TJ, Pauly D. Assessing changes in fisheries using fishers' knowledge to generate long time series of catch rates: A case study from the red sea. Ecol Soc. 2014;19(1).

35. Melo L De, Damasio A, Lopes PFM, Guariento RD. Matching Fishers ' Knowledge and Landing Data to Overcome Data Missing in Small- Scale Fisheries. PLoS One. 2015;1–13.

36. Bevilacqua AH V, Carvalho AR, Angelini R, Christensen V. More than Anecdotes : Fishers ' Ecological Knowledge Can Fill Gaps for Ecosystem Modeling. PLoS One. 2016;11(5):1–18.

37. Ogle DH, Wheeler P, Dinno A. FSA: Fisheries Stock Analysis. R package version 0.8.32. 2021.

38. Benjamini Y, Hochberg Y. Controlling the false discovery rate: a practical and powerful approach to multiple testing. J R Stat Soc Ser B. 1995;57:289–300.

39. Wei T, Simko, V. R package "corrplot": Visualization of a Correlation Matrix (Version 0.84). 2017.

40. R Core Team. R: A language and environment for statistical computing. 2019.

41. Wood SN. Generalized additive models: an introduction with R. 2006;

42. Hastie T, Tibshirani R. Generalized additive models. 1990;

43. Cockroft V. A preliminary assessment of the status of Madagascar's coastal resources, with special reference to marine mammals and reptiles. 1993.

44. Brenier A, Ferraris J, Mahafina J. Participatory assessment of the Toliara Bay reef fishery , southwest Madagascar. Madag Conserv Dev. 2011;6(2):60–7.

45. Cripps G, Gardner CJ. Human migration and marine protected areas: Insights from Vezo fishers in Madagascar. Geoforum. 2016;74(February):49–62.

46. Humber F, Andriamahaino ET, Beriziny T, Botosoamananto R, Godley BJ, Gough C, et al. Assessing the small-scale shark fishery of Madagascar through community-based monitoring and knowledge. Fish Res. 2017;186:131–43.

47. Long S, Thurlow G, Jones PJS, Turner A, Randrianantenaina SM, Gammage T, et al. Critical analysis of the governance of the Sainte Luce Locally Managed Marine Area (LMMA), southeast Madagascar. Mar Policy. 2019;(August):103691.

48. Gardner CJ, Cripps G, Prémesnil L, Dewar K, Gough C, Peabody S, et al. A decade and a half of learning from Madagascar ' s first locally managed marine area. Conserv Sci Pract. 2020;(September):1–14.

49. Lena P, Richter T, Lux M, Ratsimbazafy J, Mantilla-contreras J. The challenges of community-based conservation in developing countries — A case study from Lake Alaotra , Madagascar. J Nat Conserv. 2017;40(August):100–12.

50. Fritz-vietta NVM, Ferguson HB, Stoll-kleemann S. Conservation in a Biodiversity Hotspot : Insights from Cultural and Community Perspectives in Madagascar. In: Biodiversity hotspots. 2011. p. 209–33.

51. Cripps G. Feasibility study on the protection and management of the Barren Isles ecosystem , Madagascar. Blue Ventures Conservation Report. 2009.

52. Gough C, Thomas T, Humber F, Harris A, Cripps G, Peabody S. Vezo Fishing : An Introduction to the Methods Used by Fishers in Andavadoaka Southwest Madagascar. 2009.

53. Grenier C, Colin A, De A, Grenier C. Genre de vie vezo , pêche « traditionnelle » et mondialisation sur le littoral sud-ouest de Madagascar on the South-West coast of Madagascar. 2013;

54. Rasolofonirina R. Sea cucumbers in madagascar. In: Commercial sea cucumbers: A review for the Western Indian ocean. 2007. p. 30.

55. Eriksson H, Robinson G, Slater MJ, Troell M. Sea Cucumber Aquaculture in the Western Indian Ocean : Challenges for Sustainable Livelihood and Stock Improvement. Ambio. 2012;41:109–21.

56. Mcclanahan TR, Carlos ÆJ, White AT, Defeo ÆO. Healing small-scale fisheries by facilitating complex socio-ecological systems. Rev Fish Biol Fish. 2009;19:33–47.

57. Ikiara MM, Odink JG. Fishermen Resistance to Exit Fisheries. Mar Resour Econ. 2000;14(1999):199–213.

58. Muallil RN, Geronimo RC, Cleland D, Cabral RB, Doctor MV, Cruz-trinidad A, et al. Willingness to exit the artisanal fishery as a response to scenarios of declining catch or increasing monetary incentives. Fish Res. 2011;111:74–81.

59. Bao K, Drew JA. Traditional ecological knowledge, shifting baselines, and conservation of Fijian molluscs. Pacific Conserv Biol. 2016;21:1–7.

60. Johnson AE, Jackson JBC. Fisher and diver perceptions of coral reef degradation and implications for sustainable management. Glob Ecol Conserv. 2015;3:890–9.

61. Mcclanahan TR, Cinner JE, Abunge C, Rabearisoa A, Mahatante P, Ramahatratra F, et al. Perceived Benefits of Fisheries Management Restrictions in Madagascar. Ecol Soc. 2014;19(1):1–11.

62. Sievanen L. How do small-scale fishers adapt to environmental variability ? Lessons from Baja California , Sur , Mexico. Marit Stud. 2014;13(9):1–19.

63. Marshall NA, Marshall PA, Tamelander J, Obura D, Malleret-King D, Cinner JE. A Framework for Social Adaptation to Climate Change Sustaining Tropical Coastal Communitites and Industries. 2010.

Table 1: Survey categories and items.

**Categories**	**Items**
Socioeconomic characteristics	Age
	Fishing experience
	Main fishing gear used
	Gear ownership
	Use of marine resources
	Participation in associations
	Alternative sources of income
	Education level
	Typical monthly income
State of the fishery	Overall changes in fisheries
	Changes in distance to fishing sites
	Local extinction
	Changes in fish size
Issues and ways to address them	Causes for observed changes
	Solutions
Coping strategies	Realized changes in fishing effort
	Hypothetical changes to further decline

Table 2: Summary of fishing practices and socioeconomic characteristics by gender for the fishers of Sainte-Marie, Madagascar.

	**Fishermen (n=108)**	**Fisherwomen (n=17)**
**Fishing practices**		
Mean		
Motorized pirogues/boats	7	-
Non-motorized pirogues	90	6
Foot	3	94
Gear used[×]		
Line-fishing	80	-
Nets	50	89
Diving	28	-
Traps	16	-
Spears and sticks for octopus	2	11
Investment in expensive gear		
Yes	83	39
No	17	61
Markets[×]		
Villages	82	Irregularly
International exporting fishery	60	-
Hotels	37	-
Other collectors	24	-
**Socioeconomic characteristics**		
Education		
No formal education	60	72
Primary school	30	28

First cycle of secondary school	7	-
Second cycle of secondary school	3	-
Other sources of income		
Yes	51	22
No	49	78
Average monthly income (USD)		
	122	61
Status		
Local	98	100
Immigrant	2	-
Membership to fishing associations		
Yes	65	6
No	35	94

× As multiples answers per person are possible, total does not add up to 100.

Table 3: Observed changes to the fisheries, inferred causes , suggested solutions, and coping mechanisms for the fishers of Sainte Marie, Madagascar.

	Fishermen (n=108)	Fisherwomen (n=17)
**Changes**		
Catch volume		
Decline	93	100
Increase	2	-
No changes	5	-
Fish size		
Decline	58	NA
Increase	4	NA
No changes	38	NA
Fishing site distance		
Increase	53	-
No change	38	100
Decrease	9	-
Extinct species		
Yes	56	NA
No	44	NA
**Causes** [×]		
Local human agency		
Increased number of fishers	60	33
Net evolution	34	-
Gear diversification	21	-
Free-diving evolutions	18	11
Destructive fishing	6	-

Offshore nets	-	39
Offshore hooks	-	6
Other human agency		
Migrants	20	11
Prawn trawlers	8	-
General changes		
Socioeconomic	28	6
Divines	16	44
weather unpredictability	12	6
cyclones	12	11
Sea cucumber declines	7	11
Stochastic	7	-
Don't know	-	6
**Solutions** [×]		
Local human agency		
Marine parks	32	-
Net restrictions	26	17
Free-diving restrictions	8	-
Improved compliance	9	-
Other human agency [×]		
Restrictions for migrants	15	6
General		
Spiritual	8	28
economic	12	22
Don't know	18	33
**Coping mechanisms**		
Realized adaptation to observed changes		
Increased effort	71	-

No changes	11	100
Decreased effort	18	-
Adaptation to further hypothetical changes		
Cease	8	11
Decrease	57	33
Continue	5	11
Upgrade gear	22	-
Migrate	2	-
Don’t know	-	39

[×] As multiples answers per person are possible, total does not add up to 100.

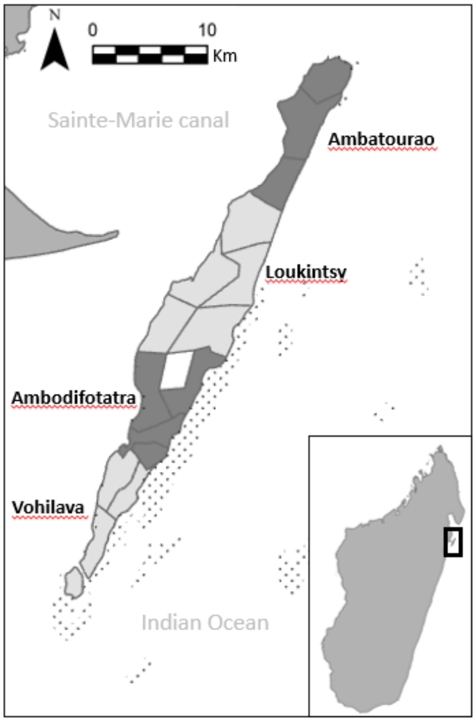

Figure 1: Map of Sainte-Marie Island, Madagascar. The boundaries of the 17 villages (contour line), the four boroughs (shades of grey), as well as coastal lagoons (dotted), are shown. The village where we could not find any fishers is blanked.

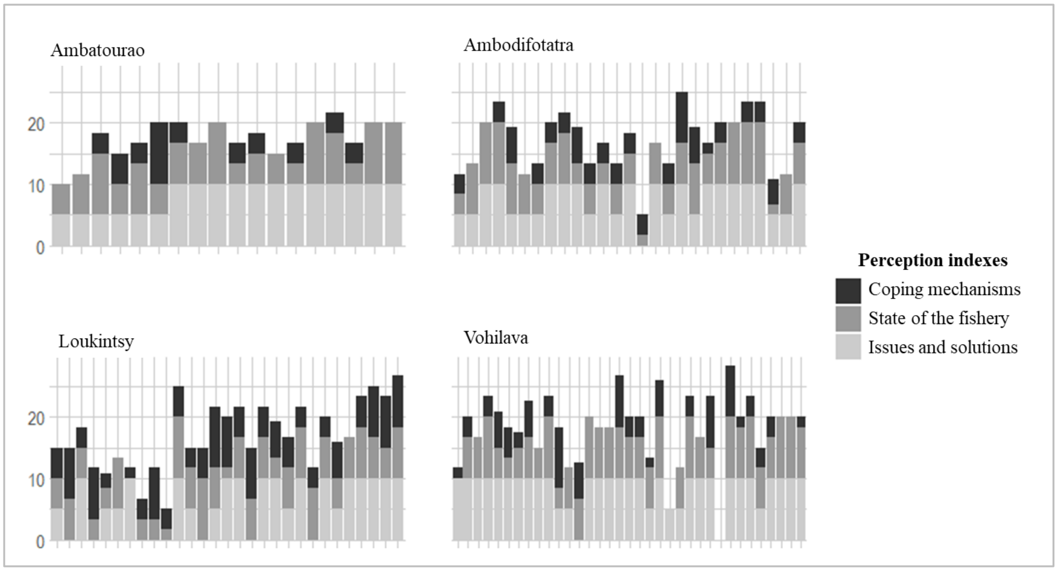

Figure 2: Perception indexes per boroughs for Sainte-Marie's fishers.

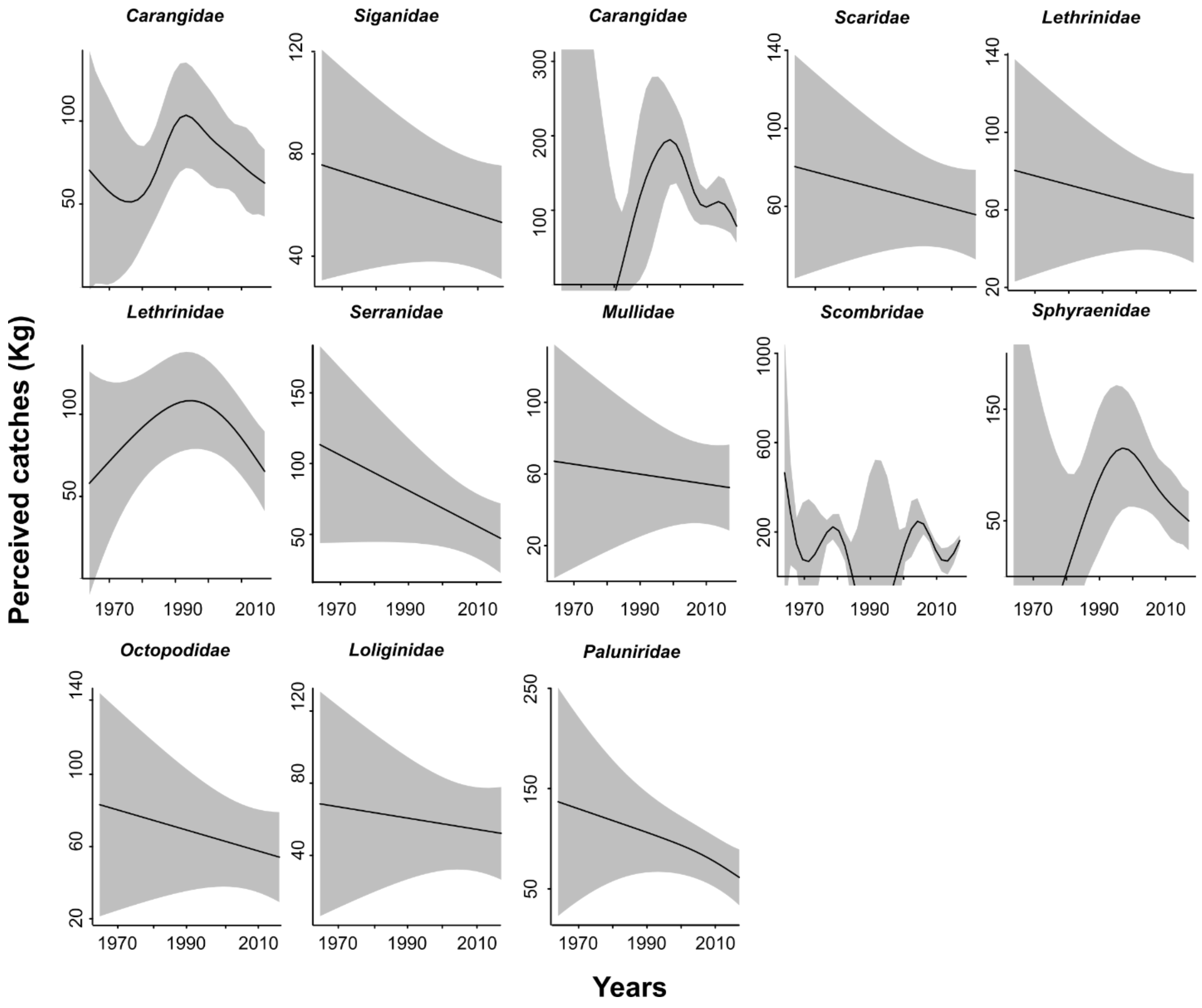

Figure 3: Fishermen perceptions of catch changes over time estimated from GAMM for 13 heavily fished species groups identified from interviews for Sainte-Marie, Madagascar. The solid line is the cubic regression spline and shaded areas represent 95% confidence intervals.

Supplementary materials

Table S1: Main species caught in Sainte-Marie, Madagascar, and proportion of the fishers targeting them by fishing method.

				**Proportion of the fishermen (%)**				
**Local name**	**Family**	**Species**	**Total**	**Hook**	**Nets**	**Diving**	**Traps**	**Foot**
Lagnoran	Carangidae	*Caranx sp., Carangoides sp.*	43	51	37	12	-	-
Femalandy	Siganidae	*Siganus sp.*	26	8	31	27	35	-
Pêche cavale	Carangidae	*Selar crumenophtalmus*	25	64	36	-	-	-
Fiambazan	Scaridae	*Scarus sp., Chlorurus sp., Cetoscarus sp., Leptoscarus sp., Calotomus sp.*	23	4	9	52	35	-
Mandrantongo	Lethrinidae	*Lethrinus harak*	21	48	33	19	-	-
Menaeliky	Lethrinidae	*Lethrinus crocineus*	20	100	-	-	-	-
Cabot	Serranidae	*Epinephelus sp., Cephalopholis sp.*	17	39	11	50	-	-
Mondrazy	Mullidae	*Parupeneus sp.*	17	24	47	18	12	-
Orita	Octopodidae	*Octopus cyanea*	15	-	-	80	-	20
Bonite	Scombridae	*Euthynnus affinis, Katsuwonus pelamis*	14	14	86	-	-	-
Saroron	Loliginidae	*Sepioteuthis sp.*	13	100	-	-	-	-
Ankasera	Sphyraenidae	*Sphyraena sp.*	11	73	27	-	-	-
Orandretsy	Palinuridae	*Palinurus sp.*	10	-	-	100	-	-

Table S2: Complete survey administrated to the fishers.

Topic	General questions
Socioeconomic and demographic characteristics	How old are you?
	How long have you been fishing for?
	What gear do you use?
	What gear do you own?
	What do you do with your catches?
	Do you belong to any association?
	Do you have other sources of income?
	What is a normal income for a month?
	What is your education level?
Perceptions	At what distance from the shore did you fish when you started?*
	At what distance from the shore do you fish now?*
	Have you noticed any general changes when you fish?
	Have you noticed the absence of known species in your catches?*
	Have you noticed the presence of novel species in your catches? *
	Have you noticed any changes in the size of the fish?*
	For 3 target species: what was a good catch over the past year (2016-2017)?* The year before the cyclone Yvan (2007-2008)?* And/or when you started to fish?*
	Has your fishing effort changed?
	What could cause the changes you observed?
	If there was a hypothetical decline of 50%, what would you do?
	What could be done to reduce the ecological changes?
Additional questions	Would you recommend your children to be fishers?
	Do you know any customary rules related to natural resource management (fady or dina)?

Are you married? Do you have children?

558 • Not applicable to the fisherwomen

Table S3: Sample sizes by gender for each of the 16 villages where fishing communities were found in Sainte-Marie, Madagascar.

**Village**	**Borough**	**Men interviewed**	**Women interviewed**	**Total interviewed**
Ambatouro	Ambatourao	9	1	10
Ambodiatafana		4	-	4
Ifotatra		5	1	6
Ambodifotatra	Ambodifotatra	8	1	9
Analaradjy		3	-	3
Ankobaoba		10	2	12
St_joseph		6	2	8
Agnafiafy	Loukintsy	4	2	6
Anivorano		4	1	5
Loukintsy		7	-	7
Maromandia		6	-	6
Sahasifotra		8	1	8
Agniribe	Vohilava	9	1	9
Ambodiforaha		7	2	7
Mahavelo		8	2	8
Vohilava		11	2	11
		109	18	127

Table S4: Assumptions underlying the indexes of perceptions

Index	Underlying assumptions
**State of the fisheries**	Perceptions of impaired ecosystem health, including declines in fish number, sizes, absence of known species, changes in fishing sites, can be interpreted as threats for local livehihoods and well-being, promoting incentives to support fishery management initiatives (5,9,56). Alternatively, ignoring the severity of the degradation -e.g. shifting baseline syndrome- does not provide any incentive to protect marine resources (59).
**Causes and Solutions**	When human behaviours (e.g. unsustainable fishing practices, habitat degradation, overfishing) are perceived as the underlying cause of observed changes (resource users or others), responses will be negotiated accordingly and typically related to fishery management. However, when causes of the decline are perceived as natural or divine, responses are less likely to have an ecological impact (9,10,60). Resource users perceiving management actions as beneficial to fishery health and the ecosystem services they provide are more likely to perceive them as legitimate and support them (1,5,12,61).
**Coping mechanisms**	Fishers who have developed strategies to cope with fishery changes have a capacity to adapt to changes (62,63). Fishers stated readiness to exit the fisheries and reduce fishing effort are an important component of their ability to adapt and manage changes related to the fisheries (Cinner et al. 2009; Muallil et al. 2011; Daw et al. 2012;Seara et al. 2016)).

Table S5: Significance levels for the perceived catch changes over time for 13 commercially important species in Sainte-Marie, Madagascar

**Group of Species**	**edf**	**p**
Caranguidae	4.6	0.06 .
Siganidae	1	0.34
Caranguidae	3.8	<0.001***
Scaridae	1.4	0.64
Lethrinidae	4.6	0.43
Lethrinidae	2.1	0.02*
Serranidae	1	0.08 .
Mullidae	1	0.68
Scombridae	6.8	<0.001***
Sphyraenidae	2.6	0.05 .
Octopodidae	1	0.4
Loliginidae	1	0.64
Paluniridae	1.3	0.06 .

Analysis S1: Perceived changes in fishing distance from the shore over time in Sainte-Marie, Madagascar.

During the interviews, fishers were asked to indicate at which distance from the shore they used to fish when they first started and now for their main fishing gear. As models including a random effect to account for two reported distance by fishers (before/now) failed to converge, we used general linear models with a poisson distribution and a log link to evaluate potential changes in fishing distance from the shore over time. As the magnitude of the perceived changes could vary by gear (sample sizes were as follow: nets=46, hook=104, fish traps=16, and free-divers=44), we included an interaction between fishing gear and time as a fixed effect. The significance of the fixed effects were assessed using likelihood ratio tests and stepwise removal of the non-significant terms.

Figure S1.1: Perceived changes in fishing distance from the shore over time by gear

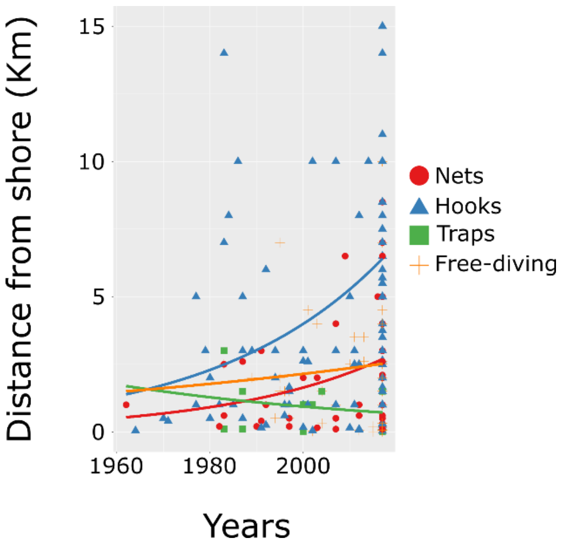

The interaction between fishing gear and time was significant (deviance=-6095, ,df=199, $p<0.001$), and results suggested that perceived fishing distance changed significantly over time and differentially amongst fishing gear. In particular, we found that the magnitude of the changes perceived were greater for those fishing with hooks, followed by nets, and then free-divers. Alternatively, our results suggested that the fishers using fish traps were now fishing closer to the shore: this contradictory result could perhaps be explained by the older age of those using fish traps as a main fishing gear.

Graphical abstract

Click here to access/download;Figure;Graphabstract.jpg

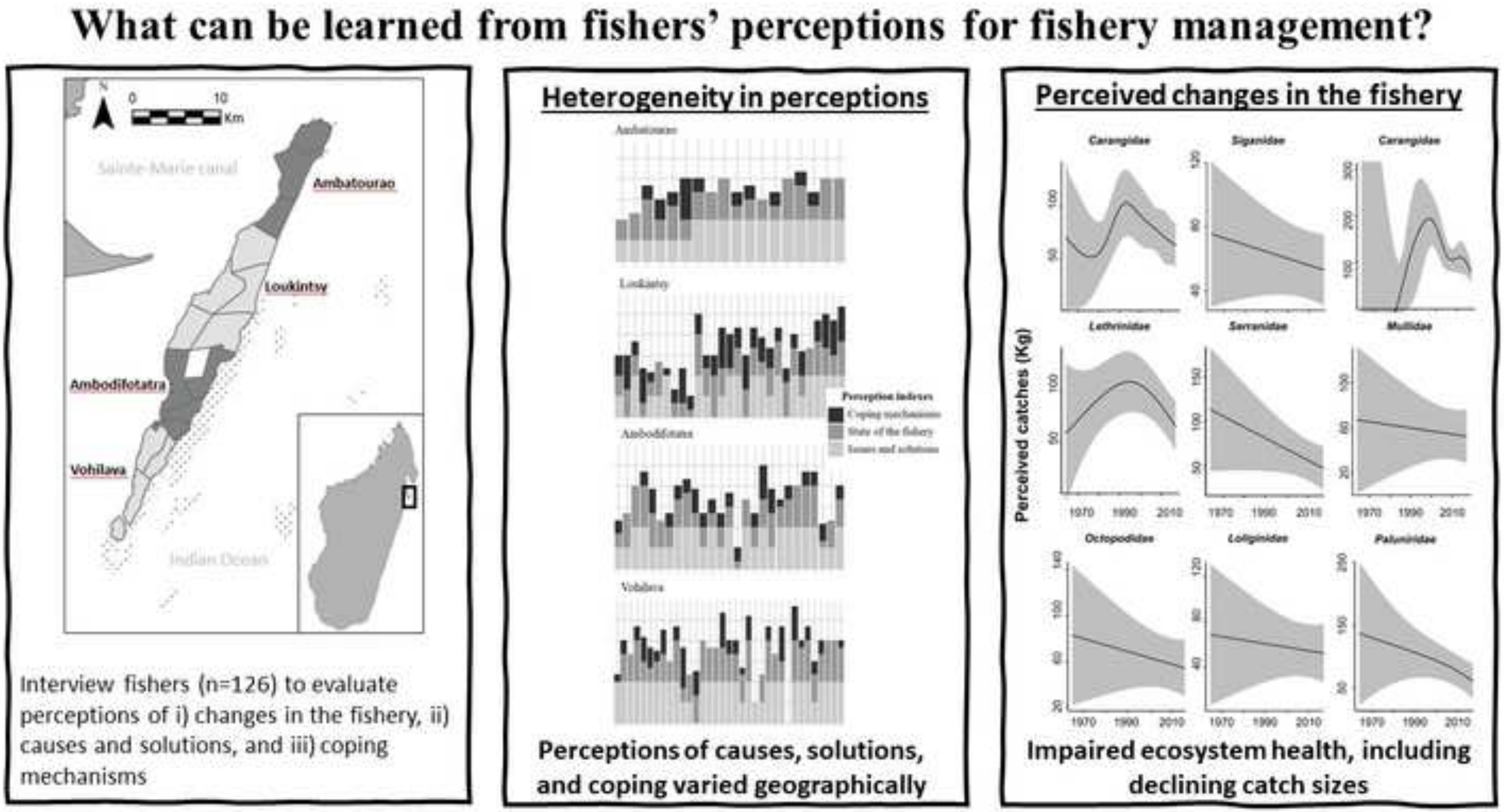

Supplement: Attachment — Submitted filename: PONE-D-21-12968_reviewer.pdf [file pone.0259792.s017.pdf]
