# Supplementary material for: What can be learned from fishers’ perceptions for fishery management planning? Case study insights from Sainte-Marie, Madagascar
Source: PLoS One. 2021 Nov 15;16(11):e0259792. doi: 10.1371/journal.pone.0259792 (PMC8592436; doi:10.1371/journal.pone.0259792)
Supplement: Attachment — Submitted filename: Reponse to reviewers.docx [file pone.0259792.s019.docx]

**Dr. Pinheiro,**

**Thank you for giving us the opportunity to further improve our manuscript** **entitled “What can be learned from fishers’ perceptions for fishery management planning? Case study insights from Sainte-Marie, Madagascar” [PONE-D-21-12968R]. We appreciate the time and effort that you and the reviewers dedicated to providing feedback on our manuscript, and found the comments provided by the reviewers helpful.**

**The two most notable changes to the manuscript, which were directly requested by the reviewers, is that we ensured that the objectives/headers were consistently organized throughout the manuscript and reduced the length of the discussion by more than 1/3 (from 2740 to 1768 words). Furthermore, we addressed most of the minor specific comments provided by the reviewers.**

**As a result of these changes, we feel that the manuscript has greatly improved. We hope that it now meets PLOS ONE’s publication criteria.**

**We thank you for your time and look forward to hearing from you,**

**The authors.**

**Journal Requirements:** *Please review your reference list to ensure that it is complete and correct. If you have cited papers that have been retracted, please include the rationale for doing so in the manuscript text, or remove these references and replace them with relevant current references. Any changes to the reference list should be mentioned in the rebuttal letter that accompanies your revised manuscript. If you need to cite a retracted article, indicate the article’s retracted status in the References list and also include a citation and full reference for the retraction notice.*

**R: To our knowledge, none of the papers cited in the manuscript have been retracted. We reviewed the reference list to ensure that it is complete and correct. As one of the reviewers suggested that we had too many references, 22 references were removed from the updated draft.**

**Reviewers’ comments:**

Note: The lines referred to in the reviewers’ comments and authors’ responses refer to the original manuscript.

Italics: *Reviewers’ comments*

**Bold: Authors’ responses and actions**

***Bold italics: edits incorporated into revised manuscript***

**Reviewer #1:**

*Lines 4-8: “The objectives of this study were two-fold. First, we examined the relationship between conservation-oriented attitudes (e.g. fishers’ propensity to suggest management restrictions), individual characteristics, and site-level characteristics for the small-scale fishers of Sainte-Marie Island, Madagascar. Second, we collated local fishers’ knowledge to understand the historical dynamics of the fishery.” and “We examined the relationship between fishers conservation-oriented attitudes (e.g. propensity to suggest management restrictions) and investigated the historical dynamic of fisheries by assessing fishers knowledge in Madagascar”. Please, note that the order of aims at the end of the introduction is different from the abstract.*

**R: We thank the reviewer for pointing this out. In the revised version of the manuscript, we made sure that the order of aims at the end of the introduction (l62) and in the abstract (l4) were consistent:**

***“First, we collated local fishers’ knowledge to characterize the long-term socio-ecological dynamics of the small-scale fishery of Sainte-Marie Island, in Madagascar. Second, we empirically assessed what individual- and site-level factors might influence support for fishery restrictions.”* (L4)**

***“First, we characterized the long-term socio-ecological dynamics of Sainte-Marie’s fishery; second, we empirically assessed what factors might influence support for fishery restrictions.”*** **(L62)**

*Line 221-226: This is a description of an obvious data organization for most of the studies; I believe this entire paragraph can be excluded.*

**R: We agree with reviewer 1. As suggested, we removed the paragraph from the revised version of the manuscript (L221-226).**

*Fig. 1. Please, add the scale of the geographical coordinate in the axis in the bottom map.*

**R: Unfortunately, we are not sure how to address this comment as the map already includes a scale bar to provide a visual indication of distance on the map. However, to further help readers locate the study area, we added the geographical coordinates of the main town in the main text of the manuscript (L101). We are more than happy to adjust the map as needed but would need more clarification about what the reviewer means.**

*Could the authors reduce the Discussion length? There are 16 paragraphs in this section, it is too long.*

**R: In the revised version of our manuscript, we reduced the length of the discussion/caveats by more than 1/3. Specifically, it was reduced from 2740 to 1808 words (18 to 13 paragraphs).**

**Reviewer #3:** 
The manuscript “What can be learned from fishers’ perceptions for fishery management planning? Case study insights from Sainte-Marie, Madagascar” has improved a lot. I’m glad to see a better explanation of the method and a broader contextualization of the study findings, as well as considerations about the study's caveats and local fisheries management.

**R: Thank you!**

However, the new version of the manuscript still needs some revision to become suitable for publication. For instance, the manuscript is considerably longer, I believe that the number of pages should be reduced. Besides, the Results and Discussion sections could be better organized, for instance, the authors could keep the same sequence when presenting objectives and subheadings in the results/discussion sections. Please, see below some suggestions.

**R: The reviewer is correct in that, as a result of addressing reviewers comments during the previous round of review, the manuscript had become significantly longer; we significantly shortened the revised version of the manuscript. In particular, we reduced the discussion/caveats section of the manuscript by more than 1/3 (from 2740 to 1768 words)** **and removed about 20% of the references (from 121 to 99). As suggested by reviewer 3, we have also improved the organization of the results and discussion and made sure that objectives and subheadings were presented in the same sequence throughout the manuscript. Please see our answers to specific comments below.**

Specific comments:
Line 4: Try to write the study’s objectives in the same way it was written throughout the manuscript. In the Introduction section they had written in a clearer way (see lines 64-66, and lines 91-92).

**R: We thank the reviewer for pointing this out. In the revised version of the manuscript, we rewrote the sentence stating the study objectives to improve clarity (L4). As suggested below, we also reorganized the objectives (L64-66 and 91-92) and ensured that they were consistently ordered in the abstract (L4) and the introduction (L62).**

***“First, we collated local fishers’ knowledge to characterize the long-term socio-ecological dynamics of the small-scale fishery of Sainte-Marie Island, in Madagascar. Second, we empirically assessed what individual- and site-level factors might influence support for fishery restrictions.”* (L4)**

***“First, we characterized the long-term socio-ecological dynamics of Sainte-Marie’s fishery; second, we empirically assessed what factors might influence support for fishery restrictions.”*** **(L62)**

Line 62: It should be the last paragraph of the Introduction. Maybe the author could transfer the current last paragraph (line 84) to line 62 but consider rewrite the sentence in lines 92 – 94 to update the order of objectives.

**R: We agree. As suggested by reviewer 3, we inserted what was the last paragraph (L84) of our previous manuscript at line 62. As a result of this change, we removed the sentence that was L92-94 and updated the order of objectives (L62) as follow;**

**“First, we characterized the long-term socio-ecological dynamics of Sainte-Marie’s fishery; second, we empirically assessed what factors might influence support for fishery restrictions. To do so, we 1) assessed perceptions related to changes in the fisheries, their causes, potential solutions, and fishers’ coping mechanisms and 2) examined whether fishers’ propensity to suggest management restrictions correlated with key factors known to influence support for conservation.”**

Line 76: This figure should be in the “Study area and context” section, after the first paragraph. Maybe it could be better to put this section in the Material and methods, before “Survey strategy”.

**R: During the previous round of review, another reviewer suggested that Figure 1 be cited and included in the introduction, where Sainte-Marie is first introduced. However, we agree with reviewer 3 that the map is better located in the Material and Method section. For this reason, we decided to move Figure 1 from the introduction (L76) back to the Study area and context section, as suggested here by reviewer 3.**

Line 202: I suggest put this section in the Supplementary material, keeping only lines 212- 218 at the end of the “Survey strategy”.

**R: We appreciate the reviewer’s insightful suggestion; however, shortening the ethical considerations section by moving parts of it to the supplementary material is not feasible. Indeed, those edits were requested by the editorial board, who specifically requested that these considerations be included in the manuscript, before the article was moved to editorial review. Nonetheless, we have tried to keep this section as short as possible in the revised paper.**

Line 220: I suggest organizing all sections of the manuscript following the same sequence of objectives. For example, the authors may start with the analysis of the temporal changes, followed by the fisher’s perceptions and then, the relationship between support for conservation and individual/site-level factors.

**R: We received conflicting advice from both reviewers regarding ways to improve the organization of this section. We decided to make the changes suggested by reviewer 1; specifically, we deleted L221-226, which described a process of data organization common to most studies. To incorporate the suggestion of reviewer 3, we also updated the heading of that section to “Statistical analysis” (L220) instead of “Analysis”. The order in which the statistical analyses are described is consistent with that of the abstract, introduction, results, and discussion (i.e. the analysis of temporal changes precedes that of the relationship between perceptions and individual/site-level factors).**

Line 278: Maybe use the word “classified” (or “categorized”) instead of codified.

**R: We replaced the word “codified” by “classified (L278).**

Line 294: This table may be presented in the Supplementary material.

**R: We appreciate the reviewer’s helpful suggestion. However, during the previous round of review, we received conflicting advice from another reviewer who suggested that the information contained in the previous version of this table be moved from the supplementary material to the main manuscript. We decided to keep the Table 1 in the main manuscript, because presenting the variables/acronyms in this table also makes it easier to interpret the acronyms figuring in Fig4.**

Line 297: Avoid discuss results in this section, it is not common to cite other studies here (just present the results). The authors should make assumptions and implications in the Discussion section only. For this comment check these lines: 307-308, 321-322, 346-349, 358-360, 393-395, 421-422, 425-426, and 481-482.

**R: We removed all of the sentences including citations from the result, as well as those where implications were discussed (e.g. L304-308, 321-322, 346-349, 358-360, 393-395, 421-422, 425-426).**

Line 339: Maybe you should keep it simple putting just “Fisher’s perceptions” and adding subheadings for each of the topics (e.g.: Changes, Causes, Solutions, and Coping mechanisms) presenting their specifics results in separated paragraphs.

**R: We thank the reviewer for this comment. As suggested, we changed the title of this section from “overview of the fisheries based on fishers’ perceptions” to “Fishers’ perceptions” (L339); we also added “Fishery Changes” (L340), “Causes” (L351), “Solutions” (L369), and “Coping mechanisms” (L378).**

Line 378: I suggest delete this subheading and keep the structure cited above.

**R: As suggested, we deleted the subheading and replaced it by “coping mechanism” (L378).**

Line 397: I suggest named this subheading “Temporal changes in catches and fishing site distance”.

**R: We thank the reviewer for this suggestion and replaced the previous subheading by “Temporal changes in catches and fishing site distance” (L397).**

Line 432: I suggest named this subheading “Fisher’s perceptions and individual and site-level factors”.

**R: We changed the subheading from “Relationship between support for conservation and various predictors” to “Relationship between fishers’ perceptions, individual-level, and site-level factors” (L432).**

Line 456 and 465: Fig. 2a and Fig. 2b?

**R: We thank the reviewer for pointing this out. We replaced Fig. 2a and Fig. 2b by Fig. 3a (L456) and Fig. 3b (L465).**

Results Section: Following my suggestion about the sequence and organization of objectives and results (comment above for line 220). I recommend organize following this way: RESULTS ◊ Fisher’s characteristics ◊ Temporal changes in catches and fishing site distance ◊ Fisher’s perceptions/Changes/Causes/Solutions/Coping mechanisms ◊ Fisher’s perceptions and individual and site-level factors.

**R: As suggested, the results (L220) are now organized under the following headers; “Fisher’s characteristics”, “Temporal changes in catch sizes and fishing site distance”, “Fishers’ perceptions” (subheaders; “fishery changes”, “Causes”, “Solutions”, “Coping mechanisms”), and “relationship between fishers’ perceptions, individual- and site-level factors”.**

DISCUSSION: I suggest reducing the number of pages of this section, the authors should be more concise with their ideas. I also recommend organizing subheadings following this way: Long-term socio-ecological dynamics in Sainte-Marie ◊ Relationship between support for management restrictions, individual, and site-level factors ◊

**R: We agree with the reviewers’ comment and reduced the length of the discussion/caveat section in the revised version of the manuscript by more than 1/3. Specifically, it was reduced from 2740 to 1768 words (from 18 to 13 paragraphs). Furthermore, as suggested by reviewer 3, we also reorganized the discussion under the following headers: “Long-term socio-ecological dynamics in Sainte-Marie”, “Relationship between support for management restrictions, individual- and site- level factors”, and “Local fishery management”.**

REFERENCES: Check for duplicates (e.g.: 27 and 86), also there are too many references, maybe the authors should reduce it.

**R: We thank the reviewer for noticing the duplicates and ensured that there was no other duplicates in the references. We added a lot of reference to the manuscript to address of the reviewers’ comments in the previous round of review, but we agree that there were too many; we therefore reduced the number of references from 121 to 99.**

Supplementary material: The authors do not need to present tables with the models' outputs. I suggest deleting the tables S5 and S7.

**R: As suggested by the reviewer, we removed Table S5 and S7 from the supplementary materials.**
